# Methylated *claudin-11* associated with metastasis and poor survival of colorectal cancer

**DOI:** 10.18632/oncotarget.21997

**Published:** 2017-10-23

**Authors:** Jinyun Li, Chongchang Zhou, Shumin Ni, Shaomin Wang, Chao Ni, Ping Yang, Meng Ye

**Affiliations:** ^1^ Department of Oncology and Hematology, Affiliated Hospital, Medical School of Ningbo University, Ningbo, Zhejiang 315000, China; ^2^ Department of Otorhinolaryngology Head and Neck Surgery, Lihuili Hospital of Ningbo University, Ningbo 315040, Zhejiang, China; ^3^ Medical School, Ningbo University, Ningbo, Zhejiang 315211, China

**Keywords:** DNA methylation, colorectal cancer, claudin-11, metastasis, progression free survival

## Abstract

As one of crucial epigenetic modification, DNA methylation plays an important role during the carcinogenesis of colorectal cancer (CRC). In the current study, we used a human genome methylation array to detect the aberrant methylation genes in CRC. We further identified the hypermethylation of *claudin-11* (*CLDN11*) and proved inverse correlation between *CLDN11* methylation and its expression in CRC. *In vitro* experiments showed debased migration ability of colonic cancer cells in accompany with the converted methylation of *CLDN11* after colonic cancer cells treated with demethylation agent, 5-aza-2’-deoxycytidine. Besides, our results also represented that hypermethylation of *CLDN11* was associated with increased metastatic potential of CRC and with low progression free survival (PFS) of CRC. In conclusion, our findings supported that the hypermethylated *CLDN11* is associated with metastasis of CRC and prognosis of poor survival of CRC.

## INTRODUCTION

Colorectal cancer (CRC) is one of the most common malignancies of gastrointestinal neoplasms, involving the malignant transformation of normal intestinal epithelial cells resulting from the accumulation of abnormal genetic and epigenetic changes [1]. CRC is diagnosed in an upward trend in China [2].

Epigenetic modifications have been documented contributing to the carcinogenesis of many human cancers [3, 4]. As one predominant modification of epigenetics, DNA methylation, plays a major role during the tumorigenesis of CRC [5]. Aberrant hypermethylation in the gene promoter region puts a damper on its transcription by recruiting methyl-cytosine-binding proteins (MBPs), generating and curtailing the binding ability of transcriptional factors [5, 6]. Thus, the hypermethylation of the gene promoter region is responsible for the crippled function of tumor suppressor genes (TSGs) and for the initiation of various human cancers, including CRC [7].

A growing list of candidate TSGs silenced by hypermethylation in CRC play important roles in CRC tumorigenesis [7, 8]. The importance of gene methylation silencing is highlighted by the growing awareness that DNA methylation can predispose to mutational changes in the early stage of CRC. For instance, O^6^-methylguanine-DNA methyltransferase (*MGMT*) functions as a remover of carcinogen-induced mistake adducts from DNA [9]. Previous studies have reported that CRC with epigenetic modified *MGMT* is prior to heritable alteration in the initial of CRC [10].

In addition, numerous studies have justified clinical values of DNA methylation in early diagnosis of CRC [11, 12]. However, few studies have investigated DNA methylation alterations occurred in the early stage of CRC that may correlate with the later progression of CRC, especially with CRC metastasis. Accordingly, identification of DNA methylation occurred in the early stage of CRC that can inform risk of subsequent metastasis of CRC has the potential to both improve our understanding of cancer progression and impact clinical treatment.

In the present study, we first identified differentially methylated genes in CRC by applying the Illumina humanmethylation450 array and then verified the hypermethylated *claudin-11* (*CLDN11*) in 125 CRC samples. *CLDN11* belongs to the claudin family, which constitutes the core components of tight junction and cell adhesion between cells. However, the association of *CLDN11* methylation with CRC progression, especially with the metastasis of CRC, was less investigated. Therefore, we assessed the methylation frequency of *CLDN11* in 125 CRC samples, and analyzed the associations between aberrantly methylated *CLDN11* and the progression of CRC, along with other clinical characteristics, such as gender, age, tumor size and differentiation. Subsequently, the effects of gene methylation exerting on the motility of CRC cells were analyzed. Through these analyses, we attempted to figure out the contribution of methylated *CLDN11* to CRC progression, and the associations between *CLDN11* methylation and progression free survival (PFS) of CRC.

## RESULTS

### Methylation array analysis of CRC tissues

**Figure 1:**
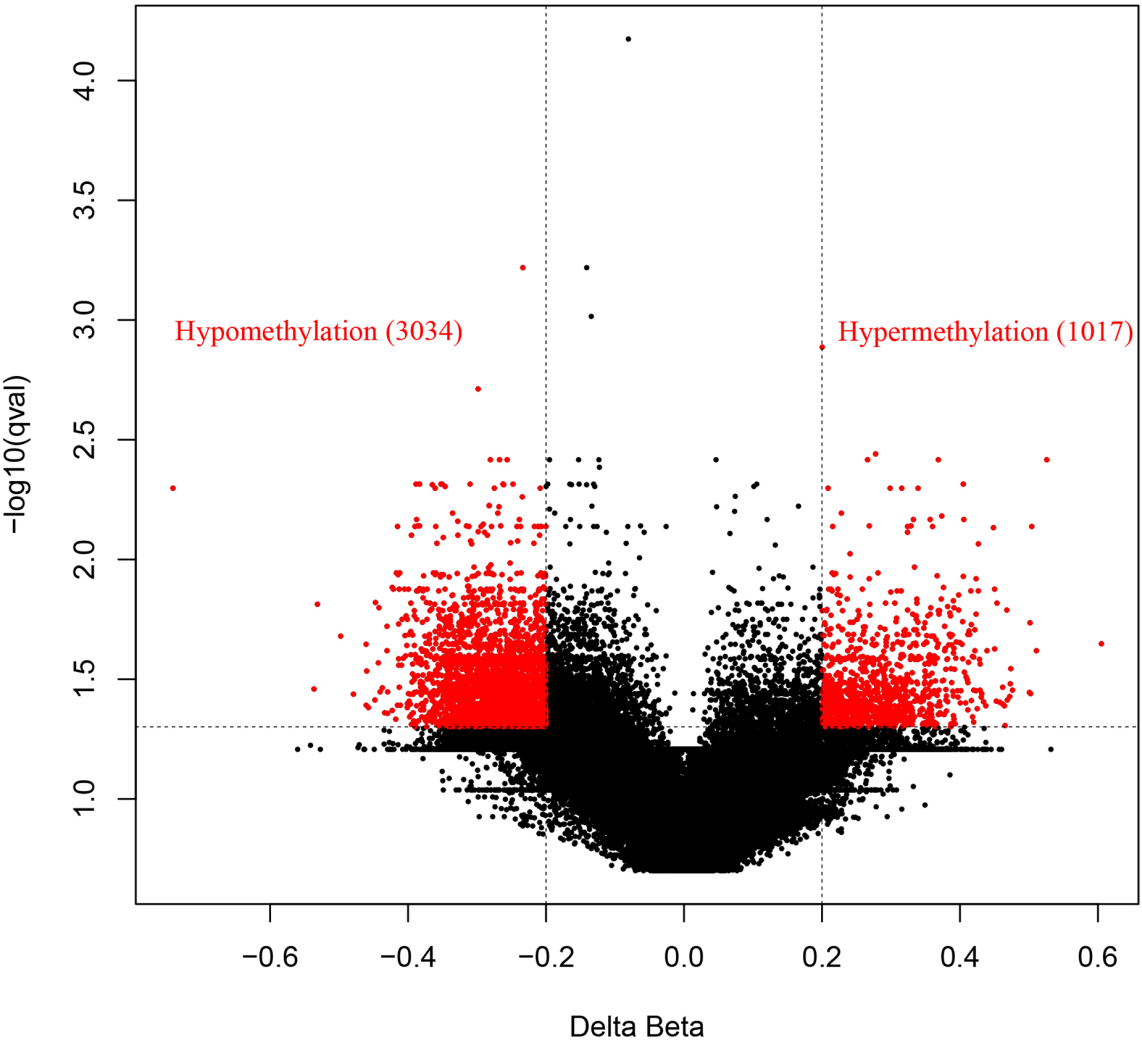
Volcano plot of methylation difference between CRC and normal tissues A total of 1017 CpG sites hypermethylated in CRC with deltaβ value > 0.2 and FDR *P* < 0.05 was represented by red point in the right side. A total of 3034 CpG sites hypomethylated in CRC with delta β value < -0.2 and FDR *P* < 0.05 was represented by red point in the left side.

**Figure 2:**
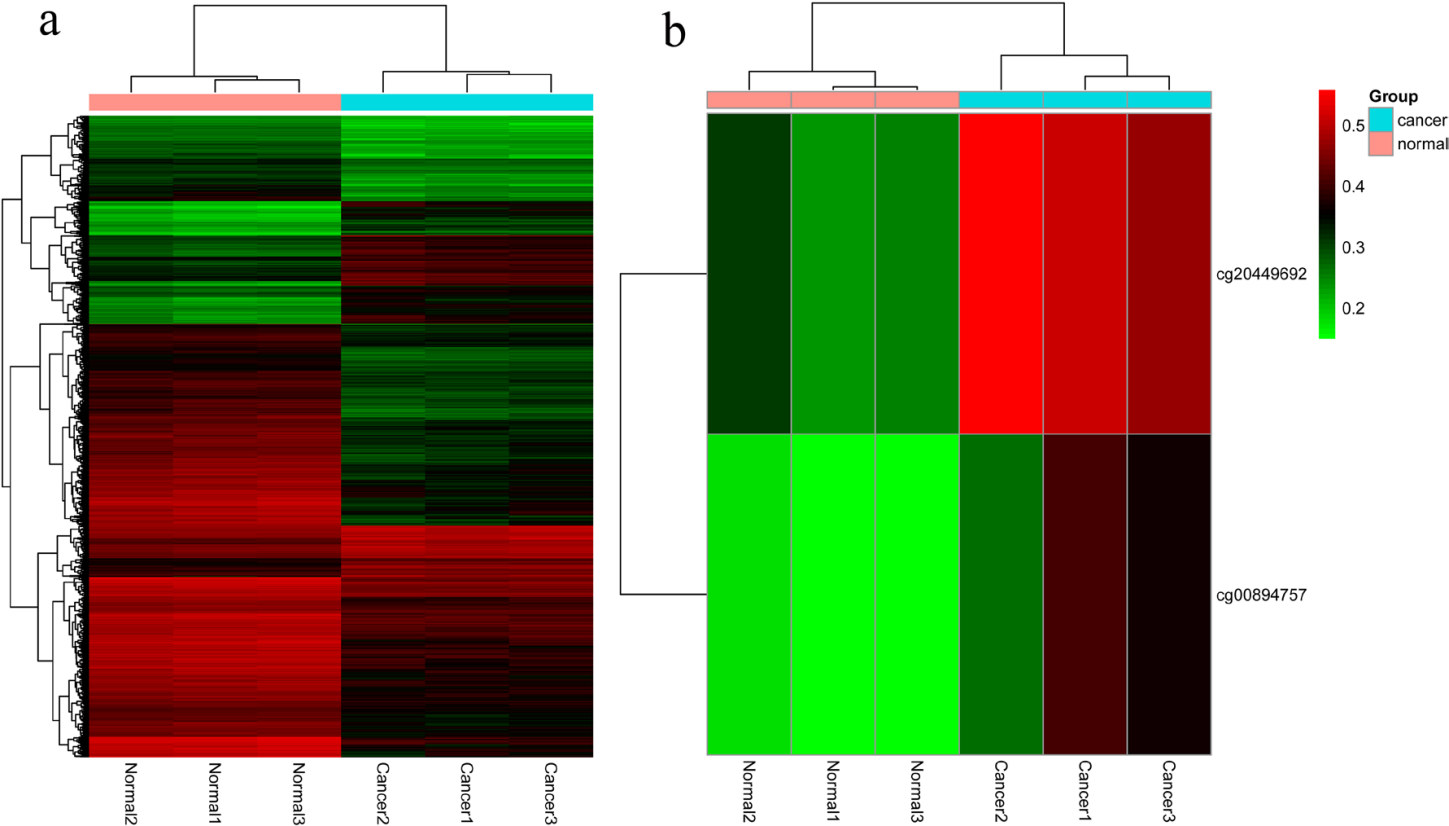
The hierarchical cluster maps of differentially methylated CpG sites in our study cohort **(a)** The hierarchical cluster maps of all the significant methylation CpG sites; **(b)** The hierarchical cluster maps of the two differentially methylated CpG sites of *CLDN11* promoter (cg20449692 and cg00894757).

To discover epigenetically-modified genes involved in CRC, we profiled genome-wide DNA methylation of three paired CRC tissues and corresponding adjacent tissues using a HM450K array. After quality control, a total of 484,698 CpG sites were analyzed. Differential methylation analysis between CRC tissues and normal tissues was identified a total of 4051 CpG sites (Supplementary Figure 1). Among which, 1017 (25%) and 3034 (75%) were hypermethylated and hypomethylated, respectively (Figure 1). These differentially methylated CpG sites were annotated to 1725 genes, among which the genes of *APC*, *MLH1* and *MGMT*, frequently reported to be methylated in CRC, were also observed in the current study (data not shown). Then, GO analysis was conducted through DAVID online database (https://david.ncifcrf.gov/) and demonstrated that the methylated genes were mainly functioned as cell adhesion and enriched in the cell adhesion molecule (CAM) pathway (Supplementary Table 1). As a result, we mainly focused on the differential methylated genes in CAM pathway for its critical role played in cell-cell adhesion and cancer metastasis. The observed maximal β value difference between CRC tissues and normal tissues was *CLDN11* gene. Figure 2 showed hierarchical cluster analysis of all differentially methylated CpG sites and the two differentially methylated CpG sites of *CLDN11* promoter (cg20449692 and cg00894757).

### Hypermethylation of *CLDN11* in CRC

**Figure 3:**
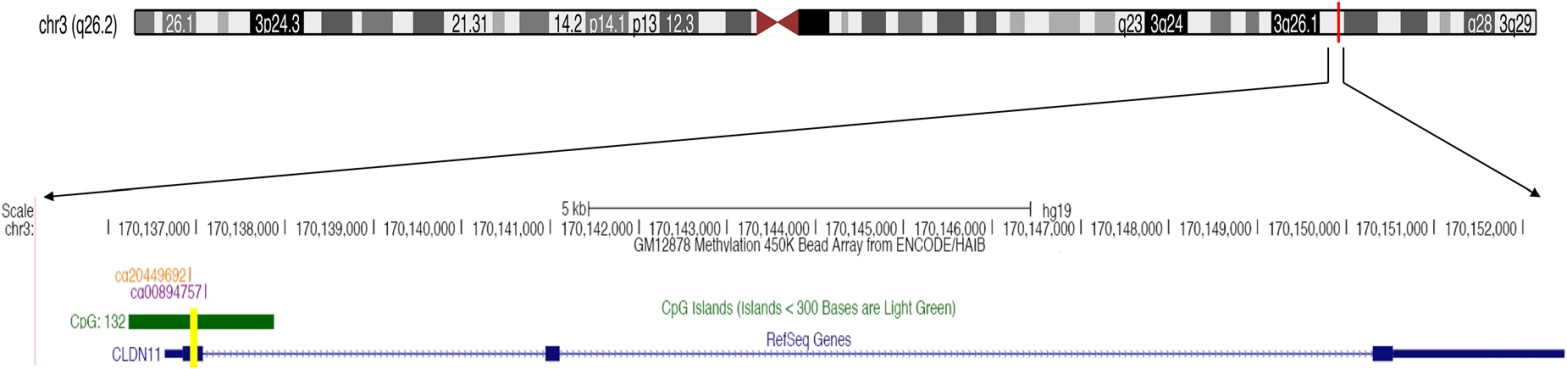
The tested fragment of *CLDN11* promoter region The yellow bar is the tested segment of *CLDN11* promoter.

**Figure 4:**
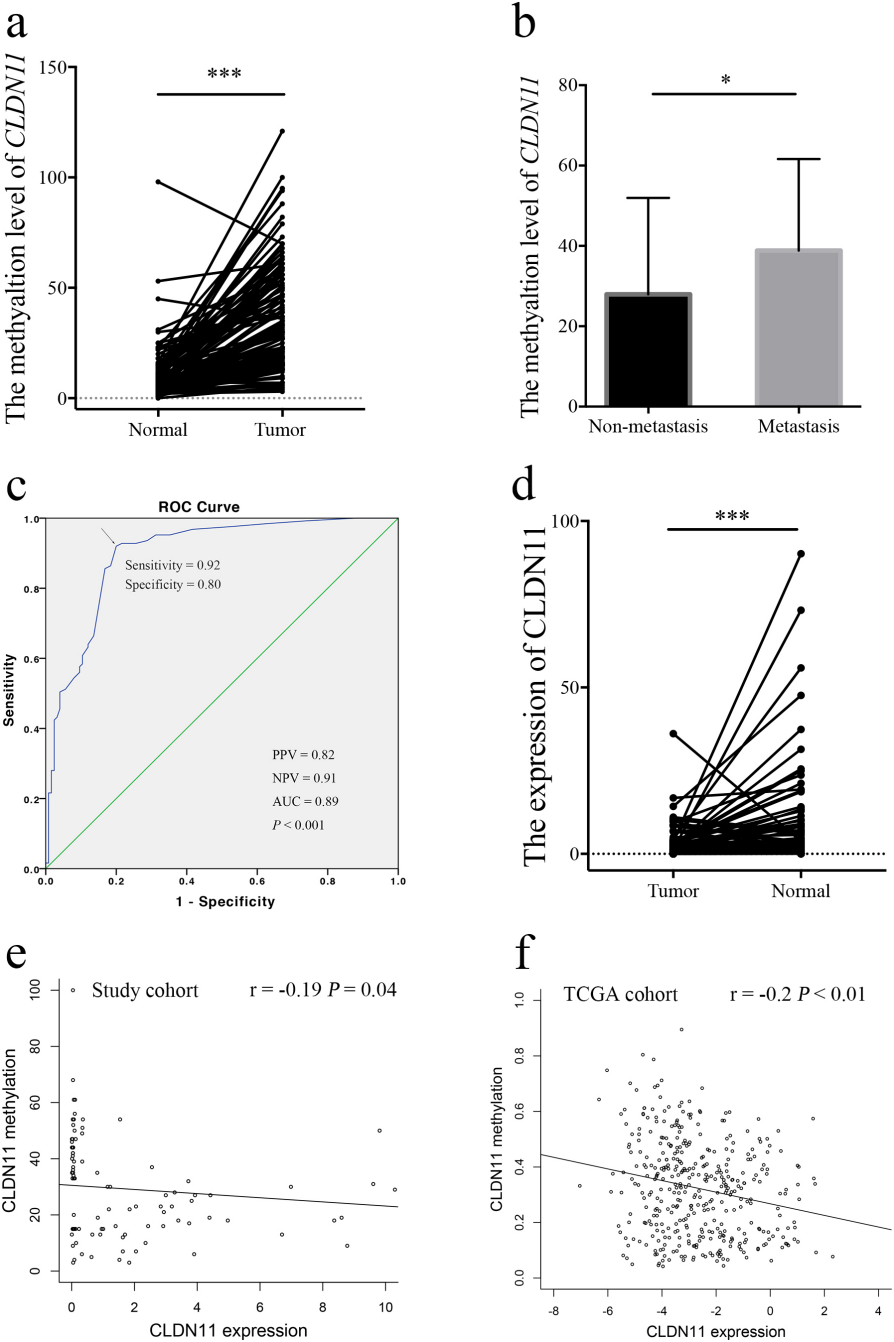
Analysis of *CLDN11* gene methylation and expression in CRC patients **(a)** Elevated methylation of *CLDN11* in CRCs; **(b)** Elevated methylation of *CLDN11* in CRCs with lymph node metastasis. **(c)** ROC curve for *CLDN11* promoter methylation. AUC: area under ROC curve; PPV: positive predict value; NPC: negative predict value; **(d)** Down-regulated expression of CLDN11 in CRC; **(e)** Inverse correlation of *CLDN11* methylation and expression in our study cohort; **(f)** Inverse correlation of *CLDN11* methylation and expression in TCGA cohort.

To verify the methylation level of *CLDN11*, the fragment of *CLDN11* promoter region was amplified for quantitative methylation polymerase chain reaction (qMSP), which neighbor the two differentially methylated CpG sites (including cg20449692 and cg00894757) (Figure 3). A total of 125 bisulphite converted DNA from formalin-fixed and FFPE CRC and normal tissues were analyzed. And the results showed the elevated methylation level of *CLDN11* in CRCs than in controls (Figure 4a). Then, methylation difference analyses based on the clinical features were performed; and we found statistically higher methylation level of *CLDN11* in CRC with lymph node metastasis when compared with CRC without metastasis (*P* = 0.01, Figure 4b). Although the methylation difference among CRCs from different clinical stage was not found, the upward trend of mean methylation frequency of *CLDN11* in adjoin with the advanced stage of CRC could be identified (Table 1). These results implied the correlation between *CLDN11* methylation and the progression of CRC.

**Table 1:** Associations of *CLDN11* methylation with clinical characteristics of CRC

Characteristics		N	Methylation% (Mean ± SD)	*P*
Gender	Male	72	33.03 ± 22.41	0.68
	Female	53	34.84 ± 25.96	
Age (y)	< 60	40	30.7 ± 22.97	0.33
	≥ 60	85	35.2 ± 24.41	
Tumor size (cm)	> 5	54	35.93 ± 26.63	0.45
	< 5	59	32.42 ± 22.07	
Location	Rectum	60	34.44 ± 26.27	0.91
	Colon	51	34.47 ± 22.40	
Differentiation	Poor	44	35.52 ± 25.37	0.052
	Moderate	79	31.87 ± 21.20	
	High	2	72.03 ± 68.65	
Lymph node metastasis	Positive	67	38.84 ± 22.81	0.01
	Negative	58	27.97 ± 23.98	
Distant metastasis	Positive	8	40.67 ± 32.33	0.41
	Negative	117	33.32 ± 23.32	
Clinical stage	I	12	21.32 ± 13.69	0.054
	II	46	29.77 ± 25.80	
	III	59	38.54 ± 21.63	
	IV	8	40.67 ± 32.33	

SD stands for standard deviation.

We also conducted a receiver operating characteristic (ROC) analysis for the diagnostic values of methylation *CLDN11* in distinguish of CRC, showing that the area under ROC curve (AUC) was 0.89 with 92% sensitivity and 80% specificity (*P* < 0.001). The positive predict value (PPV) and negative predict value were 82% and 91%, respectively (Figure 4c).

### Down-regulation of CLDN11 in CRC

Considering causal relationship between promoter hypermethylation and abated transcriptional activity, we examined the mRNA levels of *CLDN11* in 100 CRC tissues and corresponding normal tissues. Our results illustrated lower expression of *CLDN11* in CRC tissues compared with normal tissues (Figure 4d). The Pearson's analysis showed an inverse correlation between methylation and expression (r = -0.19, *P* = 0.04, Figure 4e). Besides, we abstracted methylation and expression data of *CLDN11* from TCGA (https://cancergenome.nih.gov/) (Supplementary Table 2), and the results were in accordance with our data (r = -0.21, *P* < 0.01, Figure 4f).

### High luciferase activity of pGL4-*CLDN11* plasmid

**Figure 5:**
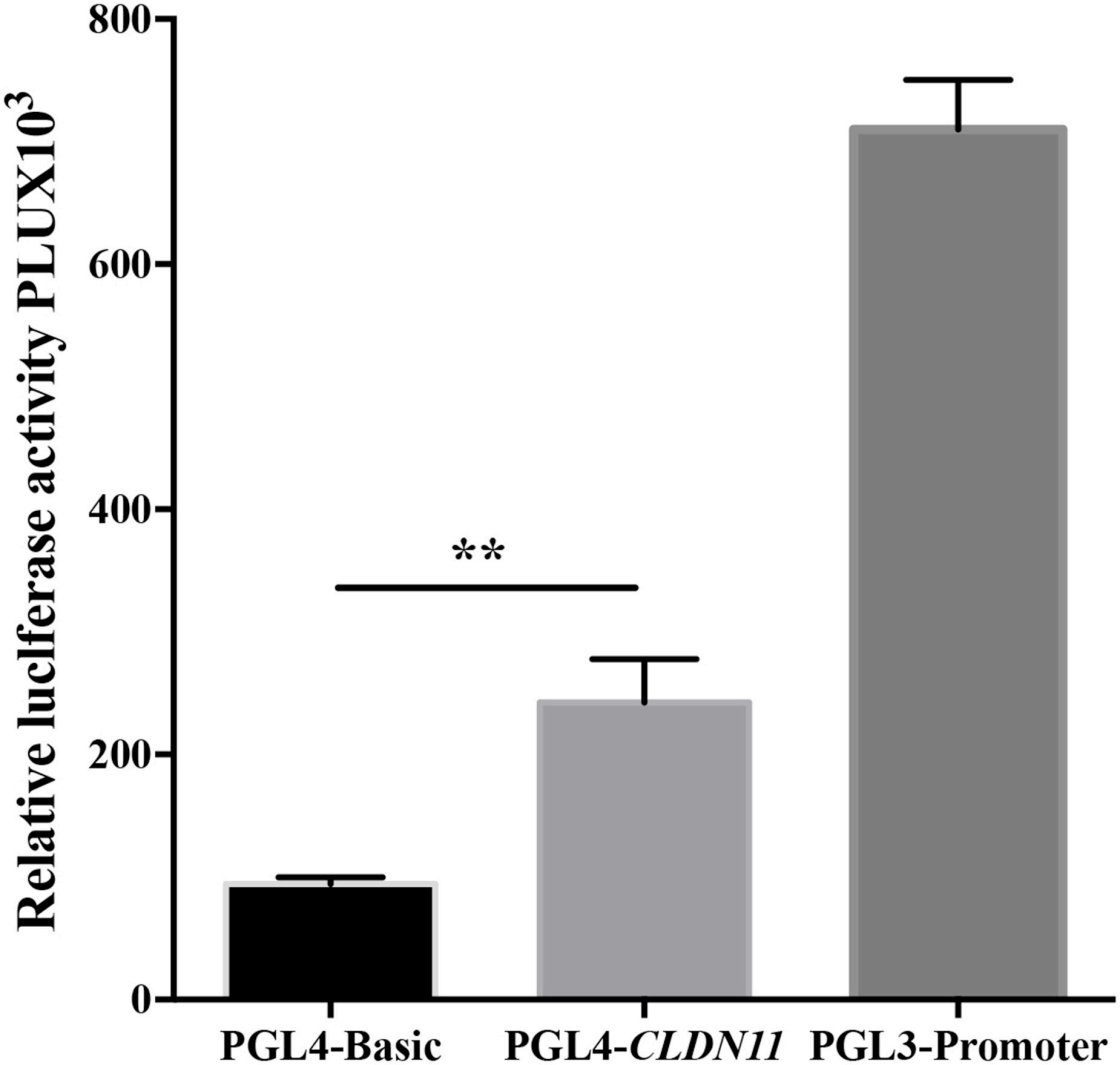
Luciferase activity analysis of fragment of *CLDN11* promoter region

To verify the transcriptional activity of the tested fragment of *CLDN11*, we performed an *in vitro* luciferase reporter assay. Our results showed that the recombinant plasmid (pGL4-*CLDN11*), containing the tested sequences of *CLDN11*, had a higher luciferase activity (Figure 5), implying that the hypermethylation of *CLDN11* promoter region might be responsible for its decreased expression.

### Differential methylation and expression of *CLDN11* in CRC cell lines

**Figure 6:**
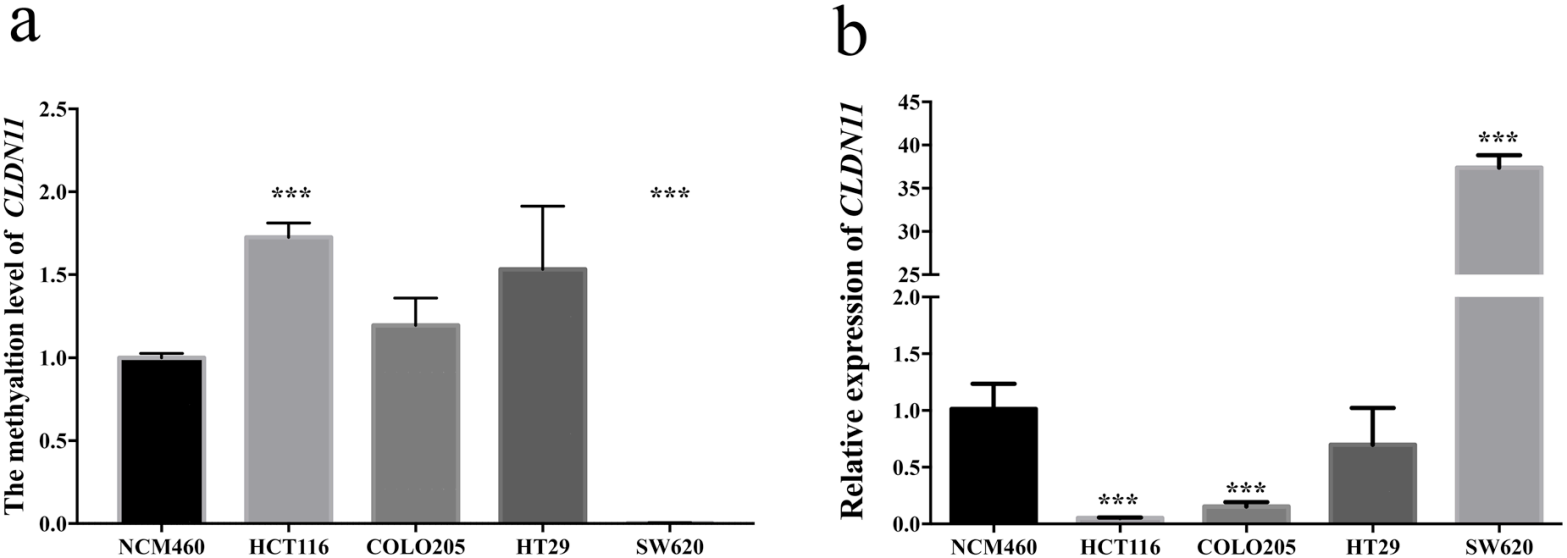
Analysis of *CLDN11* methylation and expression in CRC cell lines and normal colon cell line **(a)** Statistically elevated methylation of *CLDN11* in HCT116 and diminutive methylation of *CLDN11* in SW620; **(b)** Statistically decreased expression of CLDN11 in HCT116 and COLO205, and increased expression of CLDN11 in SW620.

Next, we checked the methylation and expression distribution of *CLDN11* in CRC cell lines (including HCT116, COLO205, SW620 and HT29) and normal colon cell line (NCM460), observing statistically elevated methylation level in HCT116, and remarkably diminutive methylation level in SW620 compared with in NCM460 (Figure 6a). Simultaneously, we found significantly lower *CLDN11* expression in HCT116 than in NCM460; and significantly higher *CLDN11* expression in SW620 was found (Figure 6b). These results showed that reduced *CLDN11* expression is associated with DNA methylation. Therefore, we selected HCT116 cell line stepping into next experiment for its characteristics with statistically high methylation level and low expression of *CLDN11*.

### Differential methylation and expression of *CLDN11* in HCT116 cells with different concentration of 5-Aza

**Figure 7:**
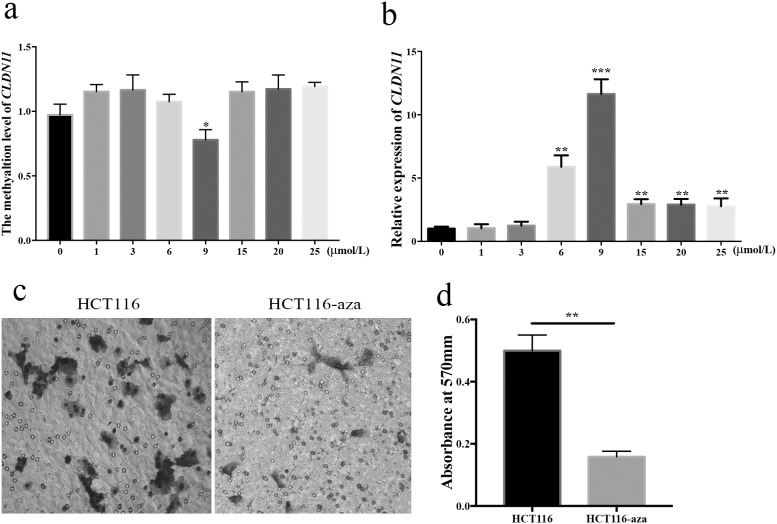
Association of *CLDN11* methylation with CRC cell migration **(a)** Methylation level of *CLDN11* in HCT116 cells treated with seven different concentration of 5-Aza; **(b)** Expression of CLDN11 in HCT116 cells treated with seven different concentration of 5-Aza; **(c)** Abated migration ability of HCT116 cells after treated with 5-Aza; **(d)** High absorbance value of eluent crystal violet solution of HCT116 cell treated with 5-Aza at 570nm.

To furtherly determine whether methylation of *CLDN11* promoter region is a potential mechanism of inactivating *CLDN11*, we measured mRNA levels of *CLDN11* in HTCT116 cells treated with 5-Aza. A total of eight different concentrations of 5-Aza were set to treat HCT116 cell line. The methylation status of HCT116 cells was assessed in the meantime. From Figure 7a and Figure 7b, we found that the methylation level of *CLDN11* was significantly reduced in cells treated with 9μM of 5-Aza (*P* = 0.04); and the restored expression of *CLDN11* was noticeable. These data implied the negative correlation between methylation and expression of *CLDN11*.

### Restored expression of *CLDN11* inhibited migration of HCT116 cells

Subsequently, we carried out a migration assay to analyze the effect of *CLDN11* methylation in CRC cells to cell migration ability, showing that the migration of HCT116 cells was dramatically decreased when *CLDN11* methylation was converted with demethylation agent (Figure 7c and Figure 7d). The Transwell results testified that hypermethylation of *CLDN11* promoter region in CRC cells committed the metastasis of cells.

### Survival analysis on TCGA cohort

**Figure 8:**
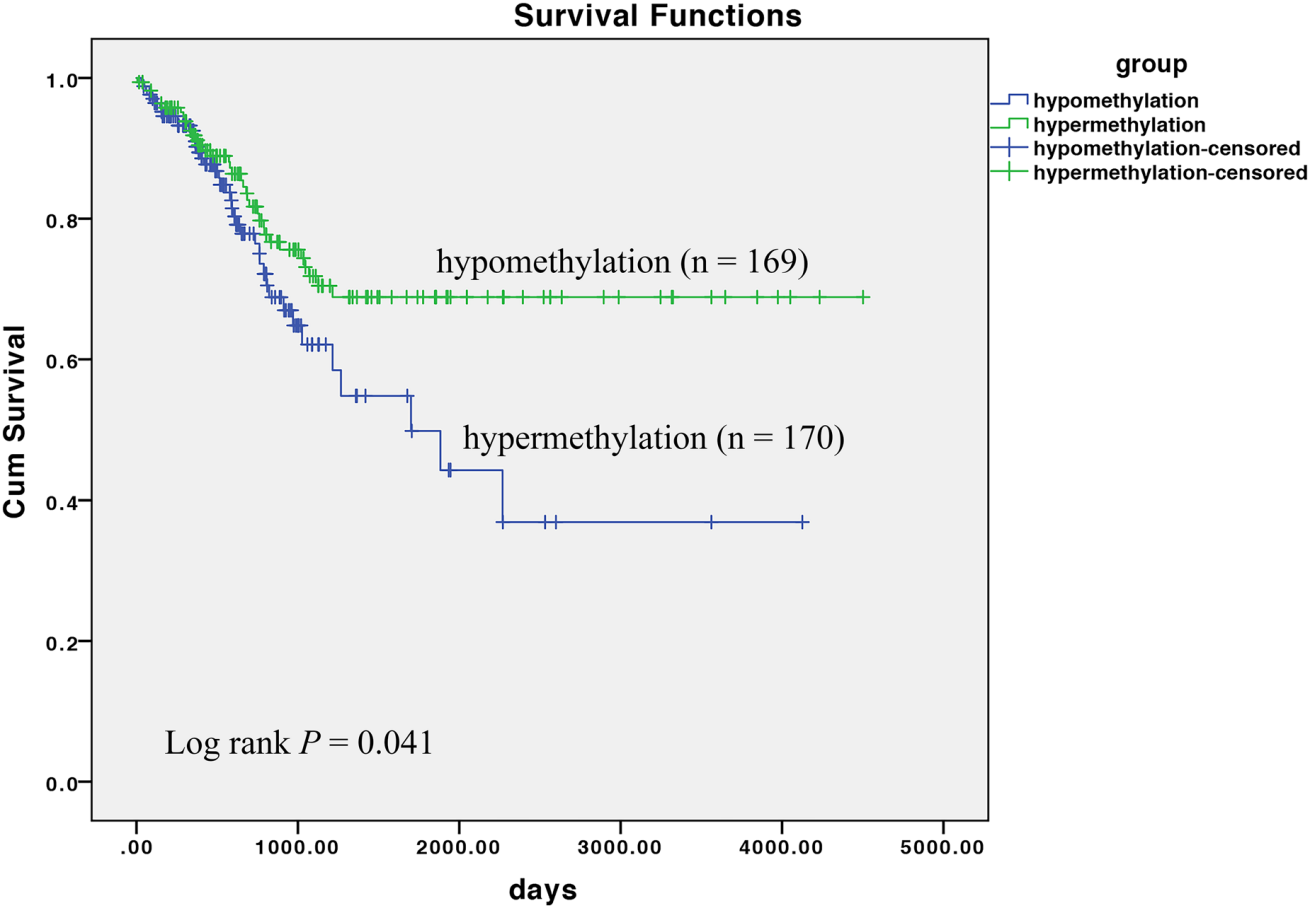
Association of *CLDN11* methylation with progression free survival (PFS) of CRC

Regrettably, we are absent of the survival data of all included patient in the current study. Therefore, to analyze the clinical implication of *CLDN11* methylation to CRC, we obtained the progression free survival (PFS) data, along with the methylation data of CRC from TCGA (Supplementary Table 3). Based on the mean methylation of two CpG sites of *CLDN11*, we classified all CRC patients as hypermethylation group (methylation data more than mean methylation) and hypomethylation group (methylation data less than mean methylation). Subsequently, we performed Kaplan-Meier survival and log-rank test, observing that patients with hypermethylation level of *CLDN11* harbored low progression free survival (PFS) (*P* = 0.041) (Figure 8), which indicated *CLDN11* methylation could serve as a useful prognosis biomarker for CRC.

## DISCUSSION

In the recent decades, outgoing studies have demonstrated that tumor cells are activated by genetic and epigenetic changes, but also routinely use epigenetic processes to render their escape from host surveillance [13]. These epigenetically changes occurred at the early stages of malignancies, and are involved the throughout tumor process including invasion [14, 15]. Both genetic and epigenetic alterations in cancer result in *de novo* acquisition of developmental programs of cell with unlimited self-renewal potential [16].

Next-generation sequencing provides a new perspective of viewing epigenetic modification. A large-scale of cancer investigations have been identified one of key player of epigenetic changes, DNA methylation across a variety of human cancers, including colorectal cancer (CRC) [17, 18], and clarified how DNA methylation influent the process of cancer. DNA methylation is a relatively stable epigenetic change, mediating the silencing of certain genes [19]. Active gene promoters harbor the absence of DNA methylation in the CpG island located in more than 60% of tumor suppressor genes. Abnormal gains of DNA methylation in the unmethylated gene promoter CpG island associated with the loss of tumor suppressor function [19], as genetic mutation. Whereas, DNA methylation occurred at early stages of malignancies, even before mutations [20]. Therefore, emerging studies have identified multiple aberrant methylation genes and implicated candidates for the development of specific markers for cancer detection and diagnosis [21, 22].

In the current study, genome-wide DNA methylation of CRC and normal control was measured by Illumina Infinium HumanMethylation450 array, targeting 485,512 CpG sites and covering 99% of RefSeq genes of the genome [23]. After analysis of the methylation array data, we observed a unanimous phenomenon of the GO analysis results of differential methylated genes between CRC and normal tissues (Figure 1 and Supplementary Table 1). Both the cellular location, biological process and KEGG pathway enrichment demonstrated methylation abnormality of genes in the cell adhesion molecules (CAMs) pathway (Supplementary Table 1). Among these genes in CAMs, the maximal differential methylated gene is *CLDN11* (Figure 2). Subsequent qMSP and RT-PCR analysis testified the high methylation of *CLDN11* in CRC, along with loss of expression (Figure 4).

*CLDN11* belongs to claudin family, of which there are 27 members. The claudins are responsible for the formation of tight junction (TJ) and cell adhesion, whose N and C-termini reside in the cytosol possesses two extracellular loop regions [24]. The cytoplasmic adaptor proteins of CLDN11 are the ZO proteins termed as ZO-1, -2 and -3, linking to the actin cytoskeleton and involving the regulation of cell motility [24]. Traditionally, TJ is intercellular structures that functions as a fence and barrier of epithelial cells, regulating and controlling the lateral diffusion of proteins within the lipid bilayer [25]. However, in the recent years, the role of TJs played in cell proliferation, transformation, and metastasis suppression, become much more intriguing. Numerous carcinomas are related with various environment stimulus and carcinogens. Therefore, our body needs a selective process to avoid these factors. Owing to the location of TJs, they are responsible for this selection and deregulation of TJs could destroy the maintenance of normal cell resulting in abnormal cellular biology, which is associated with various human disease, as well as cancers [26].

Additionally, claudin family also contributes to the adhesion between cells. Currently, the resultant of destroyed cell adhesion by the loss of claduin is critical to the metastasis of cancer cells [27, 28]. The dissociation of cancer cells from surrounding cancer cells and normal cells is the initial process of cancer cells metastasis and invasion [28]. It has been reported that recovery of various claudin proteins expression, such as *CLDN3*, *CLDN4*, and *CLDN7*, can reduce the malignant behavior of cancer cells and reduce the invasiveness in breast cancer [28]. Interestingly, claudin expression is in a tissue specific manner [29]. Victoria and his colleagues have demonstrated that one of the principal reasons of increased relatively increased expression of CLDN1 in CRC is the hypomethylation of its promoter region [30], which is also identified in our methylation array data (Supplementary Figure 2). Whereas, the different phenomenon was observed in breast cancer, hypermethylation and reduced expression of CLDN1, suggesting that the expression of CLDN1 varies in different tissues [31]. The down-regulation of *CLDN7* is found in metastatic breast cancer [32] and invasive ductal carcinomas [33]. Whereas, the transcriptional activity of *CLDN3* and *CLDN4* is increased in ovarian cancer [34]. The expression of *CLDN11* also varies differently in different tissues. It has been reported increased expression of CLDN11 in gastric cancer [35]. Nevertheless, in the current study, we observed the decreased expression of *CLDN11* in CRC. Besides, we reversed methylation level of *CLDN11* in CRC cell lines treated with 5-Aza. The deoxycytidine analog 5-aza-2’-deoxycytidine (5-Aza, also named decitabine) has been widely used as a DNA methylation inhibitor to experimentally induced gene expression, which now is postulated to have clinical activity in myelodysplastic syndrome (MDS), chronic myelogeneous leukemia (CML), acute myelogenous leukemia(AML) [36–38]. Interestingly, the effect of demethylation and restored expression of CLDN11 is significant when CRC cells were treated at low-dose of 5-Aza, rather than at high-dose. It was believed that low-dose schedules of 5-Aza could be served as antitumor drug to inhibitor DNA methylation and to reactivate gene expression [39, 40]. However, the covalent enzyme-DNA adduct, rather than DNA demethylation, is main reason for the toxicity of 5-Aza at high dose in cultured cell lines [41], which means the effect of demethylation and inversion of gene is slight when the tumor cells is cultured at high dose of 5-Aza. This might be one of the principle reason why slight demethylation and increased expression of CLDN11 in CRC cells treated with high dose of 5-Aza in our study. As shown in Figure 7, the results implied that DNA methylation contributes to the deduced expression of CLDN11 in CRC. However, researches on a correlation between *CLDN11* methylation and CRC have been limited so far. In our study, our results and TCGA cohort showed a correlation between *CLDN11* methylation and the metastasis and poor survival of CRC (Figure 4 and Figure 8). An *in vitro* experiment also portrayed the aggrandized migration ability of human colonic cancer cells with hypermethylation of *CLDN11* (Figure 7).

Previous studies have elaborated the expression of *CLDN3* and *CLDN4* effect on the metastatic ability of breast cancer cells [42]. In cervical cancer cells, *CLDN1* overexpression could promote invasion and metastasis [43]. On the other hand, claudin-low breast cancers have potential to metastasis and chemoresistant [44].

In a conclusion, our investigation shows the epigenetic modification mechanism of loss of CLDN11 expression in CRC. Besides, our results and TCGA cohort datasets represent the association between *CLDN11* methylation and metastasis and unsatisfactory survival of CRC, which implies the potential of methylated *CLDN11* in prognosis of CRC.

## MATERIALS AND METHODS

### Patients

At an initial discovery set, DNA from three matched patients CRC with corresponding normal tissues was applied the genome-wide DNA methylation analysis on HumanMethylation450 platform. All patients were enrolled in Gastrointestinal Surgery in Affiliated Hospital of Ningbo University and diagnosed by pathology. Before operation and tissue collection, all patients were informed and signed the consent forms. The adjacent normal tissue was at least 5 cm away from the tumor. All the samples were stored in liquid nitrogen at -80°C after surgery. On an independent validation set, a total of 125 paired formalin-fixed and paraffin-embedded (FFPE) CRC and cutting edge tissues were collected from Diagnostic Pathology Central of Ningbo. All patients were pathological diagnosed.

### DNA and total RNA extraction

Genomic DNA was extracted by QIAamp DNA Mini Kit or DNA FFPE Tissue Kit (Qiagen, Hilden, Germany) as recommended by the manufacturer. Total RNA from FFPE tissues were isolated using RNA FFPE Tissue Kit (Qiagen, Hilden, Germany) according to the manufacturer's protocol. The concentration and quality of DNA, as well as of RNA, was measured by ultramicro nucleic acid ultraviolet tester (NANODROP 1000, Wilmington, USA).

### Bisulfite treatment

Extracted DNA was bisulphite-converted with ZYMO EZ DNA Methylation-Gold Kit according to the manufacturer's protocol (Zymo Research, Orange, CA, USA). The bisulphite-modified DNA was resuspended in 10 μl of TE buffer for the following methylation analysis.

### Illumina humanmethylation450 beadchip

The bisulphite converted DNA of three paired CRC and adjacent normal tissues were used for hybridization on the HumanMethylation450 BeadChip (HM450K, Illumina, San Diego, CA, USA). The bead array data were read by Illumina's Genome Studio program (Methylation Module). And the raw methylation data was exported from Genome Studio and analyzed using Bioconductor *minfi* packages.

### Quantitative methylation polymerase chain reaction (qMSP)

Bisulphite converted DNA as described above was amplified by fluorescence-based real-time quantitative methylation-specific PCR (qMSP) using SYBR Green Master Mix (Roche Diagnostics) with Roche 480 II RT-PCR instrument (Roche Diagnostics). The qMSP primers for *CLDN11* were designed by MethPrimer (www.urogene.org/methprimer/) [45]. Each reaction involved 1x PCR buffer and 0.5 μM forward primer and reverse primer in a total volume of 10 μl. The PCR reaction were conducted as follows: 95°C for 10 min, amplification for 45 cycles of 95°C for 20 sec, 60°C for 30 sec and 72°C for 30 sec. The primer of *ACTB* were simultaneously amplified of each sample as internal control [46] and the full methylated DNA from healthy person (Qiagen, Hilden, Germany) served as a positive methylation control. The methylation levels were calculated as the difference in *C*_t_ value between *CLDN11* and *ACTB* using the following formula: 2^[ΔΔ*C*t (Positive control)- ΔΔ*C*t (Samples) ]^ ×100 [46], and ΔΔ*C*_t_ = *C*_t_ (*CLDN11*)- *C*_t_ (*ACTB*).

### Reverse transcription-polymerase chain reaction (RT-PCR)

The cDNA was generated using the Reverse Transcription Kit (Promega, Madison city, WI, USA). Real-time RT-PCR method was performed to assess the expression level of *CLDN11* with SYBR Green Master Mix (Roche Diagnostics) on a Roche 480 II RT-PCR instrument (Roche Diagnostics). The relative expression of *CLDN11* in each sample was calculated with the 2^-ΔΔ*C*t^ method, that is -ΔΔ*C*_t_ = *C*_t_ [(*CLDN11*)-*C*_t_ (*GAPDH*) (normal samples)]-[*C*_t_ (*CLDN11*)-*C*_t_ (*GAPDH*) (tumor samples)], in which the *GAPDH* was used as internal control [47]. And the same method was used to determine the relative expression levels of *CLDN11* of all the cell lines in the current study. Each sample was measured three times. The sequences of primers in the current study were listed in Table 2.

**Table 2:** Sequences of qMSP and RT-PCR primers

Characteristics		Sequence
qMSP	*CLDN11*-Forward	5'-ATAAGTTGATAGGAGAATCGAATCG-3'
	*CLDN11*-Reverse	5'-AACGAAAATAACCCTAAACACGTT-3'
	*ACTB*-Forward	5'-TGGTGATGGAGGAGGTTTAGTAAGT-3'
	*ACTB*-Reverse	5'-AACCAATAAAACCTACTCCTCCCTTAA-3'
RT-PCR	*CLDN11*-Forward	5'-TATGTGGAGTGAGTGGGCCA-3'
	*CLDN11*-Reverse	5'-AACCAGATGGTGGCAACAAC-3'
	*GAPDH*-Forward	5'-ACCCACTCCTCCACCTTTGAC-3'
	*GAPDH*-Reverse	5'-TGTTGCTGTAGCCAAATTCGTT-3'

### Cell culture

Human colorectal cancer cell lines HCT116, COLO205, HT29 and SW620, as well as human normal colon epithelial cell line NCM460 were obtained from Shanghai Institute of Biochemistry and Cell Biology, Chinese Academy of Sciences (Shanghai, China). Cells were cultured at 37°C in a humidified atmosphere of 5% CO_2_ with RPMI-1640 Medium (HyClone, Logan, Utah) containing 10% fetal bovine serum (FBS) (ExCell Biology, Shanghai, China) with 50 U/ml penicillin and 50g/ml streptomycin (HyClone, Logan, Utah). Exponentially growing cells were used for experiments.

### Construction of recombinant plasmids

A fragment of *CLDN11* promoter region was synthesized which included the tested segment of *CLDN11* by qMSP. The recombinant plasmid termed as pGL4-*CLDN11* concatenated the fragment of *CLDN11* and pGL4 Basic vector (Promega, Madison, WI, USA). The pRL-SV40 vector (Promega) with Ranilla luciferase gene was applied as an internal control and the pGL3-Promoter vector (Promega) was used as a positive control in the current study.

### Transfection and reporter gene activity assay

The human HEK293T cells in exponential growth phase were cultured in 24-well plates (5×10^4^/well) in 500μl RPMI-1640 with 10% FBS. After 12h culture, cells of 70% attachment were transfected with pGL4-*CLDN11*, pGL4-Basic, pRL-SV40 and pGL3-Promoter vector using the Lipofectamin 2000 reagent according to the transfection protocol recommended by Invitrogen (Invitrogen Corp., Carlsbad, CA, USA). After 24 h of 293T cells transfection, Renilla and firefly luciferase activity was detected by SmartSpec Plus spectrophotometer (Bio-Red, Hercules, CA, USA). Reporter gene activity was measured by following the manufacturer's protocol (Dual-Luciferase Reporter Assay Systems, Promega).

### Treatment of HCT116 cells with 5-aza-2’-deoxycytidine

The human colorectal cancer cell lines HCT116 were cultured into a 24-well plate and exposed to eight different concentrations of 5-aza-2’-deoxycytidine (5-Aza) for 3 days (including 0μM, 1 μM, 3 μM, 6 μM, 9 μM, 15 μM, 20 μM and 25 μM) (Sigma-Aldrich, St. Louis, MO, USA). The cells treated with 5-Aza were harvested and used for detection of *CLDN11* methylation and expression.

### Transwell migration assays

Transwell migration assay was performed with 24-well matrigel-coated chambers (8-μm pore size) (Corning Costar, NY, USA). Briefly, cells were serum-starved for 12h and subsequently dissociated with trypsin. After washed by PBS for twice, cells were resuspeded in serum-free medium and a total of 5×10^4^ cells were added to the upper chamber adding complete medium on the bottom well. After 48h seeding, medium was aspirated and the cells that were unable to migrate were removed from the surface of the upper chamber. Therefore, the migrating cells were fixed by formaldehyde and stained by crystal violet solution. Images of three different fields of view were captured from each membrane under a microscope. The membranes were dissolved with 10% acetic acid, and absorbance was detected by SpectraMax 190 (Molecular DevicesA). Each assay was independently performed three times.

### Extraction data from TCGA portal

From The Cancer Genome Atlas (TCGA, https://cancergenome.nih.gov/) portal, we downloaded the Illumina HumanMethylation 450K methylation frequencies and RNA-seq expression of *CLDN11*, as well as the progression free survival (PFS) data of 395 CRC patients. For methylation data of *CLDN11*, we abstracted the level 3 methylation data of the two CpG sites (including cg20449692 and cg00894757), which were identified differentially methylated in our CRC samples. Based on the mean methylation frequencies of these two CpG sites of *CLDN11*, 339 CRC patients with PFS data were classified as hypermethylation group and hypomethylation group, and then the effect of *CLDN11* methylation level to CRC outcome was analyzed. A total of 394 CRCs with both methylation and expression data of *CLDN11* were used to verify the association between expression and methylation.

### Statistical analysis

The methylation data from Illumina humanmethylation450K were analyzed using R 3.1.2 software (https://www.r-project.org/). The raw data were imported into R software using Bioconductor *minfi* [48]. Probes with a detection *P* value > 0.01 and those measuring SNPs or mapping in sex chromosomes were removed. Raw data was background corrected by subtracting control probes, normalized using subset-quantile within array normalization (SWAN) [49] to correct the discrepancies between type I and type II probes, and then computed methylated and unmethylated intensities. The methylation levels of CpG sites were calculated as β-value (β = intensity [methylated]/intensity [methylated+unmethylated+100]). Subsequently, M-values were calculated by transforming β-value for the following formula: M = log_2_(β/(1-β)) [50]. Then, the differential methylated CpG-sites (using M-values) between CRC and normal controls were identified. Benjamini-Hochberg false-discovery rate (FDR) method adjusted *P* value of each probe was calculated. FDR adjusted *P* value less than 0.05 and the delta β value either more than 0.2 (termed as hypermethylation) nor less than -0.2 (termed as hypomethylation) were used as the cut-off values, highlighting by red points in the volcano plot. Subsequently, genes of all the hypermethylation probes were annotated based on the reference genes of Illumina arrays, and performed the Gene Ontology (GO) using web-based DAVID (https://david.ncifcrf.gov/).

The following analyses were carried out using Statistical Program for Social Sciences (SPSS) software 13.0 (SPSS, Inc., Chicago, IL, USA), and represented by GraphPad software (InStat). The paired samples and two independent samples *t*-test were applied under the appropriate condition to compare the difference of methylation and expression, luciferase activity, and absorbance rate in our study cohort. ROC analysis was used to study the diagnostic power of gene methylation in distinguishing of CRC. The correlation of methylation and expression was computed by the Pearson rank test, representing using R software. In TCGA cohort, we collected the PFS data of CRC, analyzed the impact of methylation level on the PFS by Kaplan-Meier method, plotted Kaplan-Meier survival curves and compared using the log-rank test. Statistical significance was defined as *P* < 0.05.

## SUPPLEMENTARY MATERIALS FIGURES AND TABLES









## Abbreviations

CRC: colorectal cancer
*CLDN11*: *claudin-11*
5-Aza: 5-aza-2’-deoxycytidine
PFS: progression free survival
MBP: methyl-cytosine-binding protein
TSG: tumor suppressor gene
*MGMT*: O^6^-methylguanine-DNA methyltransferase
FFPE: formalin-fixed and paraffin-embedded
HM450K: HumanMethylation450 BeadChip
qMSP: fluorescence-based real-time quantitative methylation-specific polymerase chain reaction
RT-PCR: reverse transcription-polymerase chain reaction
FBS: fetal bovine serum
TCGA: The Cancer Genome Atlas
FDR: false-discovery rate
GO: Gene Ontology
SPSS: Statistical Program for Social Sciences
CAM pathway: cell adhesion molecule pathway
ROC: receiver operating characteristic
AUC: area under ROC curve
PPV: positive predict value
NPC: negative predict value
TJ: tight junction.
