# Supplementary material for: Methylated claudin-11 associated with metastasis and poor survival of colorectal cancer
Source: Oncotarget. 2017 Oct 23;8(56):96249–62. doi: 10.18632/oncotarget.21997 (PMC5707097; doi:10.18632/oncotarget.21997)
Supplement: Supplementary file 1 [file oncotarget-08-96249-s001.pdf]

Methylated *claudin-11* associated with metastasis and poor survival of colorectal cancer

SUPPLEMENTARY MATERIALS

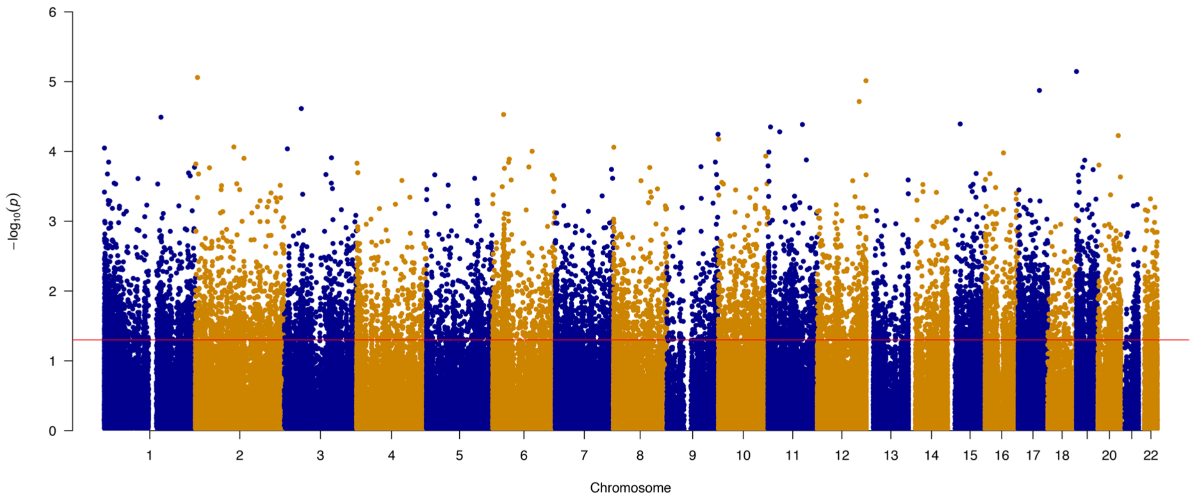

Supplementary Figure 1: All differential methylated CpG sites of our methylation array displayed by Manhattan plot.

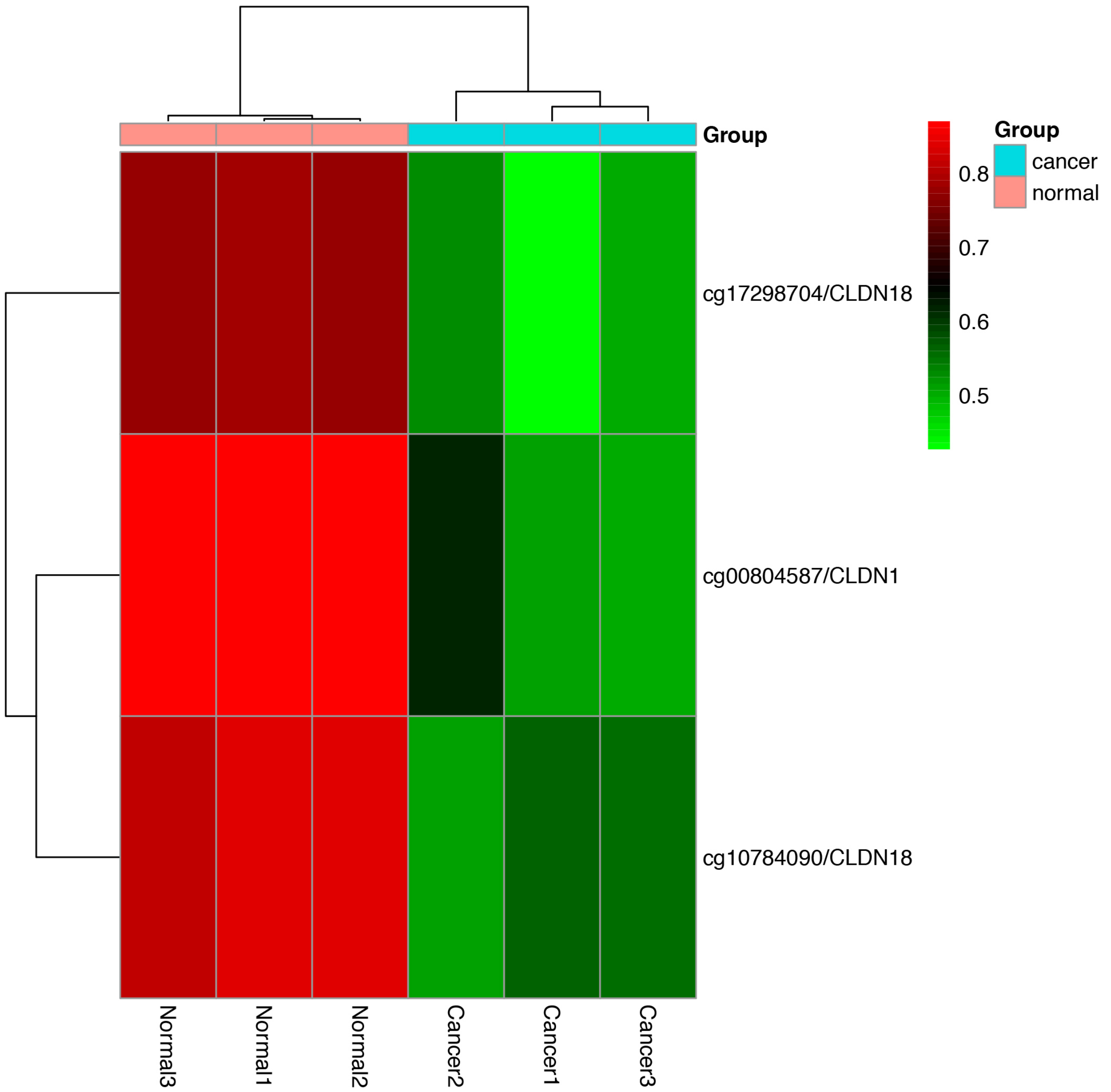

Supplementary Figure 2: Three differential hypomethylated CpG sites of *CLDN1* and *CLDN18* in our study cohort.

Supplementary Table 1: GO enrichment of differentially methylated genes

See Supplementary File 1

Supplementary Table 2: Methylation and expression dataset of *CLDN11* of 394 CRC patients from TCGA

See Supplementary File 2

Supplementary Table 3: Progression free survival (RFS) data of 339 CRC patients from TCGA

See Supplementary File 3
