# Supplementary material for: Methylated claudin-11 associated with metastasis and poor survival of colorectal cancer
Source: Oncotarget. 2017 Oct 23;8(56):96249–62. doi: 10.18632/oncotarget.21997 (PMC5707097; doi:10.18632/oncotarget.21997)
Supplement: Supplementary file 2 [file oncotarget-08-96249-s002.docx]

**Supplementary Table 1: GO enrichment of differentially methylated genes**

|  | ID | Term | Count (n) | Percent (%) | Benjamini |
| --- | --- | --- | --- | --- | --- |
| Biological process | GO:0007155 | **cell adhesion** | 79 | 5.038265306 | 2.78528E-07 |
|  | GO:0007268 | chemical synaptic transmission | 41 | 2.614795918 | 0.010007981 |
|  | GO:0007411 | axon guidance | 31 | 1.977040816 | 0.008279276 |
| Cellular location | GO:0005886 | plasma membrane | 453 | 28.89030612 | 1.66641E-14 |
|  | GO:0005578 | proteinaceous extracellular matrix | 50 | 3.18877551 | 4.68743E-06 |
|  | GO:0009986 | cell surface | 77 | 4.910714286 | 7.25377E-05 |
|  | GO:0016021 | integral component of membrane | 484 | 30.86734694 | 0.000120231 |
|  | GO:0005887 | integral component of plasma membrane | 158 | 10.07653061 | 0.000332035 |
|  | GO:0030054 | **cell junction** | 63 | 4.017857143 | 0.001390066 |
|  | GO:0031225 | anchored component of membrane | 23 | 1.466836735 | 0.004251734 |
|  | GO:0031012 | extracellular matrix | 44 | 2.806122449 | 0.003890963 |
|  | GO:0045202 | synapse | 31 | 1.977040816 | 0.004112356 |
| Molecular function | GO:0005509 | calcium ion binding | 107 | 6.823979592 | 7.13615E-08 |
|  | GO:0001077 | transcriptional activator activity, RNA polymerase II core promoter proximal region sequence-specific binding | 40 | 2.551020408 | 0.003391636 |
|  | GO:0000977 | RNA polymerase II regulatory region sequence-specific DNA binding | 33 | 2.104591837 | 0.060571222 |
|  | GO:0005201 | extracellular matrix structural constituent | 16 | 1.020408163 | 0.046981895 |
|  | GO:0008013 | beta-catenin binding | 18 | 1.147959184 | 0.03853239 |
|  | GO:0005262 | calcium channel activity | 16 | 1.020408163 | 0.045007304 |
| KEGG | hsa04510 | Focal adhesion | 36 | 2.295918367 | 0.007570659 |
|  | hsa04911 | Insulin secretion | 20 | 1.275510204 | 0.006583423 |
|  | hsa04514 | **Cell adhesion molecules (CAMs)** | 23 | 1.020408163 | 0.043399088 |
|  | hsa04918 | Thyroid hormone synthesis | 16 | 1.147959184 | 0.038156209 |
|  | hsa04970 | Salivary secretion | 18 | 1.147959184 | 0.035156272 |
|  | hsa04512 | ECM-receptor interaction | 18 | 1.849489796 | 0.031917119 |
|  | hsa04020 | Calcium signaling pathway | 29 | 1.275510204 | 0.037656118 |
|  | hsa05146 | Amoebiasis | 20 | 1.147959184 | 0.037373322 |
|  | hsa05032 | Morphine addiction | 18 | 3.25255102 | 0.036889229 |
|  | hsa05200 | Pathways in cancer | 51 | 1.211734694 | 0.038078475 |
|  | hsa04723 | Retrograde endocannabinoid signaling | 19 | 0.956632653 | 0.04278407 |
|  | hsa05412 | Arrhythmogenic right ventricular cardiomyopathy (ARVC) | 15 | 1.147959184 | 0.040719368 |
|  | hsa04713 | Circadian entrainment | 18 | 1.977040816 | 0.042833123 |
|  | hsa04015 | Rap1 signaling pathway | 31 | 1.020408163 | 0.044928154 |
|  | hsa04925 | Aldosterone synthesis and secretion | 16 | 1.275510204 | 0.042585762 |
|  | hsa04724 | Glutamatergic synapse | 20 | 1.466836735 | 0.048731776 |
