# Supplementary material for: Methylated claudin-11 associated with metastasis and poor survival of colorectal cancer
Source: Oncotarget. 2017 Oct 23;8(56):96249–62. doi: 10.18632/oncotarget.21997 (PMC5707097; doi:10.18632/oncotarget.21997)
Supplement: Supplementary file 3 [file oncotarget-08-96249-s003.docx]

**Supplementary Table 2: Methylation and expression dataset of *CLDN11* of 394 CRC patients from TCGA**

| sample | methylation | expression |
| --- | --- | --- |
| TCGA-AY-6196-01 | 0.07705 | 2.30928 |
| TCGA-AZ-6599-11 | 0.09215 | 1.69298 |
| TCGA-AD-6901-01 | 0.33895 | 1.65648 |
| TCGA-AF-2690-01 | 0.3587 | 1.61128 |
| TCGA-DC-6156-01 | 0.5742 | 1.58338 |
| TCGA-D5-6534-01 | 0.2836 | 1.06928 |
| TCGA-F4-6809-01 | 0.40035 | 1.05558 |
| TCGA-A6-6782-01 | 0.33655 | 1.02268 |
| TCGA-F5-6464-01 | 0.1305 | 1.02208 |
| TCGA-F4-6569-01 | 0.405 | 1.00978 |
| TCGA-A6-2675-11 | 0.18185 | 0.910783 |
| TCGA-AZ-6598-11 | 0.1397 | 0.891583 |
| TCGA-CM-5348-01 | 0.20085 | 0.886383 |
| TCGA-AA-3663-11 | 0.11745 | 0.671883 |
| TCGA-AA-3489-01 | 0.1796 | 0.656083 |
| TCGA-AZ-6600-11 | 0.1224 | 0.620383 |
| TCGA-AZ-4323-01 | 0.31855 | 0.586283 |
| TCGA-AD-6964-01 | 0.12405 | 0.577283 |
| TCGA-G4-6302-01 | 0.3037 | 0.555683 |
| TCGA-AG-3731-11 | 0.1471 | 0.477783 |
| TCGA-A6-2685-01 | 0.11005 | 0.442883 |
| TCGA-AF-2687-01 | 0.4576 | 0.393683 |
| TCGA-F4-6703-01 | 0.30425 | 0.364683 |
| TCGA-F4-6857-01 | 0.2254 | 0.354883 |
| TCGA-A6-A5ZU-01 | 0.53005 | 0.297883 |
| TCGA-F5-6812-01 | 0.2504 | 0.281083 |
| TCGA-A6-2671-11 | 0.17545 | 0.272383 |
| TCGA-A6-2686-11 | 0.1505 | 0.100683 |
| TCGA-G4-6626-01 | 0.06235 | 0.0370826 |
| TCGA-F5-6571-01 | 0.47665 | -0.0210174 |
| TCGA-AZ-6601-11 | 0.13945 | -0.0426174 |
| TCGA-AA-3697-11 | 0.16515 | -0.0687174 |
| TCGA-G4-6299-01 | 0.45375 | -0.101017 |
| TCGA-A6-6142-01 | 0.39155 | -0.107417 |
| TCGA-DM-A28H-01 | 0.5015 | -0.111917 |
| TCGA-F4-6459-01 | 0.375 | -0.128217 |
| TCGA-CM-6167-01 | 0.12255 | -0.159717 |
| TCGA-A6-A567-01 | 0.11825 | -0.234117 |
| TCGA-A6-6651-01 | 0.4075 | -0.241017 |
| TCGA-G5-6572-02 | 0.16725 | -0.250017 |
| TCGA-F4-6807-01 | 0.3914 | -0.302817 |
| TCGA-DT-5265-01 | 0.49055 | -0.360517 |
| TCGA-F4-6463-01 | 0.34685 | -0.386217 |
| TCGA-AA-3660-11 | 0.1753 | -0.395517 |
| TCGA-EI-6917-01 | 0.50155 | -0.400117 |
| TCGA-AG-3725-11 | 0.17815 | -0.419117 |
| TCGA-AG-4021-01 | 0.57275 | -0.432917 |
| TCGA-5M-AAT6-01 | 0.1063 | -0.450017 |
| TCGA-D5-6536-01 | 0.10315 | -0.451317 |
| TCGA-CM-5344-01 | 0.37055 | -0.525617 |
| TCGA-AH-6643-01 | 0.43785 | -0.583117 |
| TCGA-F5-6813-01 | 0.35475 | -0.600617 |
| TCGA-F5-6864-01 | 0.4636 | -0.659917 |
| TCGA-D5-6541-01 | 0.19895 | -0.700017 |
| TCGA-F5-6702-01 | 0.2836 | -0.717317 |
| TCGA-EI-7004-01 | 0.2691 | -0.719217 |
| TCGA-EI-6885-01 | 0.4277 | -0.724217 |
| TCGA-AZ-6607-01 | 0.44605 | -0.816417 |
| TCGA-EI-6507-01 | 0.4272 | -0.816817 |
| TCGA-F4-6461-01 | 0.5292 | -0.839117 |
| TCGA-G5-6572-01 | 0.27545 | -0.849617 |
| TCGA-A6-5662-01 | 0.07355 | -0.876217 |
| TCGA-AA-3655-11 | 0.13825 | -0.892517 |
| TCGA-AA-3713-11 | 0.12825 | -0.938617 |
| TCGA-A6-2679-11 | 0.15965 | -0.954117 |
| TCGA-D5-5538-01 | 0.34145 | -0.956017 |
| TCGA-CA-6717-01 | 0.34205 | -0.967217 |
| TCGA-A6-2680-11 | 0.09245 | -0.976317 |
| TCGA-A6-6654-01 | 0.3784 | -1.00972 |
| TCGA-A6-5664-01 | 0.3231 | -1.06812 |
| TCGA-G4-6627-01 | 0.2841 | -1.08092 |
| TCGA-CM-5868-01 | 0.10285 | -1.08472 |
| TCGA-A6-2684-01 | 0.2560165 | -1.08912 |
| TCGA-3L-AA1B-01 | 0.12435 | -1.11902 |
| TCGA-A6-5667-11 | 0.1129 | -1.14402 |
| TCGA-EI-6509-01 | 0.17685 | -1.16032 |
| TCGA-AH-6644-01 | 0.33095 | -1.17802 |
| TCGA-D5-5541-01 | 0.35015 | -1.20122 |
| TCGA-CM-6168-01 | 0.3125 | -1.31422 |
| TCGA-CM-6169-01 | 0.24525 | -1.33422 |
| TCGA-DM-A1HA-01 | 0.48675 | -1.36742 |
| TCGA-A6-6649-01 | 0.35405 | -1.39022 |
| TCGA-CK-6748-01 | 0.45725 | -1.40012 |
| TCGA-A6-2684-11 | 0.13835 | -1.40342 |
| TCGA-F4-6855-01 | 0.3529 | -1.40982 |
| TCGA-A6-2685-11 | 0.14335 | -1.41762 |
| TCGA-D5-6928-01 | 0.30985 | -1.43142 |
| TCGA-F5-6810-01 | 0.13055 | -1.45872 |
| TCGA-DM-A28G-01 | 0.11415 | -1.47262 |
| TCGA-AD-6899-01 | 0.29965 | -1.47522 |
| TCGA-CA-5254-01 | 0.51305 | -1.47612 |
| TCGA-A6-2686-01 | 0.59765 | -1.50302 |
| TCGA-F4-6704-01 | 0.36495 | -1.54942 |
| TCGA-CM-6676-01 | 0.42725 | -1.55262 |
| TCGA-A6-5657-01 | 0.32395 | -1.55962 |
| TCGA-F4-6805-01 | 0.0656 | -1.57002 |
| TCGA-G4-6628-01 | 0.5021 | -1.60142 |
| TCGA-CM-4751-01 | 0.3849 | -1.60942 |
| TCGA-D5-6929-01 | 0.31455 | -1.61222 |
| TCGA-CM-6165-01 | 0.40795 | -1.61602 |
| TCGA-SS-A7HO-01 | 0.46455 | -1.61862 |
| TCGA-AA-3662-01 | 0.24205 | -1.62732 |
| TCGA-AZ-6605-01 | 0.32945 | -1.63342 |
| TCGA-DY-A1H8-01 | 0.3354 | -1.63752 |
| TCGA-CK-5916-01 | 0.5944 | -1.64112 |
| TCGA-F5-6863-01 | 0.30685 | -1.64872 |
| TCGA-A6-2675-01 | 0.16905 | -1.68262 |
| TCGA-CA-6719-01 | 0.5669 | -1.70082 |
| TCGA-A6-2682-01 | 0.34315 | -1.71112 |
| TCGA-DC-6158-01 | 0.1338 | -1.71392 |
| TCGA-DC-6682-01 | 0.3533 | -1.71652 |
| TCGA-AZ-5403-01 | 0.1516 | -1.72002 |
| TCGA-NH-A8F7-06 | 0.04375 | -1.72602 |
| TCGA-CM-6163-01 | 0.06845 | -1.73892 |
| TCGA-A6-2682-11 | 0.154 | -1.75312 |
| TCGA-G4-6294-01 | 0.56735 | -1.76902 |
| TCGA-G4-6314-01 | 0.2737 | -1.78962 |
| TCGA-A6-4105-01 | 0.3636 | -1.79282 |
| TCGA-D5-6922-01 | 0.19385 | -1.80002 |
| TCGA-CM-6680-01 | 0.32975 | -1.80422 |
| TCGA-AG-3731-01 | 0.37895 | -1.81552 |
| TCGA-CM-6162-01 | 0.08495 | -1.82392 |
| TCGA-AA-3712-11 | 0.164 | -1.84852 |
| TCGA-G4-6317-02 | 0.0994 | -1.85542 |
| TCGA-CI-6624-01 | 0.14745 | -1.86652 |
| TCGA-F5-6811-01 | 0.07825 | -1.87642 |
| TCGA-D5-5539-01 | 0.38875 | -1.87692 |
| TCGA-EI-6513-01 | 0.09315 | -1.89762 |
| TCGA-CL-5917-01 | 0.40235 | -1.91572 |
| TCGA-AG-3732-01 | 0.17795 | -1.94532 |
| TCGA-BM-6198-01 | 0.0875 | -1.94612 |
| TCGA-AD-6965-01 | 0.30705 | -1.94742 |
| TCGA-DY-A1DF-01 | 0.06115 | -1.96542 |
| TCGA-AH-6549-01 | 0.37975 | -1.96912 |
| TCGA-A6-6781-01 | 0.3562 | -2.03342 |
| TCGA-NH-A8F8-01 | 0.455 | -2.06832 |
| TCGA-A6-A565-01 | 0.2074 | -2.06862 |
| TCGA-G4-6625-01 | 0.2503 | -2.10762 |
| TCGA-A6-6138-01 | 0.08115 | -2.13112 |
| TCGA-5M-AAT4-01 | 0.16485 | -2.13862 |
| TCGA-CM-4747-01 | 0.28145 | -2.16062 |
| TCGA-CM-6679-01 | 0.36695 | -2.17842 |
| TCGA-F5-6465-01 | 0.1417 | -2.20022 |
| TCGA-D5-6898-01 | 0.0899 | -2.21492 |
| TCGA-G4-6298-01 | 0.2753 | -2.21542 |
| TCGA-D5-6930-01 | 0.4595 | -2.25292 |
| TCGA-AG-4022-01 | 0.16805 | -2.25402 |
| TCGA-AF-A56K-01 | 0.2465 | -2.29862 |
| TCGA-CM-5862-01 | 0.36305 | -2.30992 |
| TCGA-CI-6619-01 | 0.4633 | -2.31162 |
| TCGA-A6-A566-01 | 0.41045 | -2.34012 |
| TCGA-AF-4110-01 | 0.3494 | -2.36152 |
| TCGA-DY-A1DD-01 | 0.267 | -2.36252 |
| TCGA-AZ-4616-01 | 0.3722 | -2.36972 |
| TCGA-DY-A1DE-01 | 0.1939 | -2.39212 |
| TCGA-F4-6460-01 | 0.0772 | -2.39442 |
| TCGA-CM-5349-01 | 0.35475 | -2.40842 |
| TCGA-CA-6718-01 | 0.40575 | -2.42192 |
| TCGA-AZ-4684-01 | 0.24385 | -2.43622 |
| TCGA-AZ-6600-01 | 0.26065 | -2.45772 |
| TCGA-DC-6683-01 | 0.40165 | -2.47292 |
| TCGA-DC-6154-01 | 0.1473 | -2.47872 |
| TCGA-G4-6297-01 | 0.4371 | -2.48082 |
| TCGA-DM-A1D0-01 | 0.08255 | -2.49392 |
| TCGA-AA-3506-01 | 0.16585 | -2.51222 |
| TCGA-D5-6531-01 | 0.68385 | -2.51672 |
| TCGA-CI-6621-01 | 0.4332 | -2.53232 |
| TCGA-NH-A6GA-01 | 0.17525 | -2.54012 |
| TCGA-DM-A1D6-01 | 0.5974 | -2.58482 |
| TCGA-CM-6678-01 | 0.5177 | -2.58532 |
| TCGA-DY-A0XA-01 | 0.29115 | -2.59582 |
| TCGA-CM-5860-01 | 0.60155 | -2.60432 |
| TCGA-AY-A69D-01 | 0.1495 | -2.60442 |
| TCGA-EI-6514-01 | 0.134 | -2.61272 |
| TCGA-AG-3592-01 | 0.3136 | -2.63232 |
| TCGA-AF-A56L-01 | 0.3719 | -2.64502 |
| TCGA-AD-5900-01 | 0.55385 | -2.66512 |
| TCGA-AH-6547-01 | 0.26315 | -2.66682 |
| TCGA-CM-6164-01 | 0.1193 | -2.72192 |
| TCGA-CI-6620-01 | 0.11045 | -2.72962 |
| TCGA-NH-A50V-01 | 0.41155 | -2.76252 |
| TCGA-AZ-6603-01 | 0.2992 | -2.76802 |
| TCGA-A6-5660-01 | 0.116 | -2.78622 |
| TCGA-DM-A1D7-01 | 0.2912 | -2.80532 |
| TCGA-D5-6923-01 | 0.2336 | -2.81492 |
| TCGA-CA-5797-01 | 0.0742 | -2.82852 |
| TCGA-D5-6927-01 | 0.1153 | -2.82892 |
| TCGA-DC-5869-01 | 0.39535 | -2.83832 |
| TCGA-AF-3911-01 | 0.06565 | -2.84882 |
| TCGA-EI-6884-01 | 0.3732 | -2.86722 |
| TCGA-DC-4745-01 | 0.2479 | -2.91432 |
| TCGA-AF-6655-01 | 0.26075 | -2.91532 |
| TCGA-D5-6926-01 | 0.2602 | -2.93372 |
| TCGA-A6-5659-01 | 0.58965 | -2.93422 |
| TCGA-F5-6814-01 | 0.57695 | -2.95152 |
| TCGA-NH-A5IV-01 | 0.4891 | -2.97342 |
| TCGA-AZ-6601-01 | 0.39365 | -2.98322 |
| TCGA-EI-6883-01 | 0.63645 | -2.98582 |
| TCGA-AD-6548-01 | 0.361 | -2.99082 |
| TCGA-CM-6170-01 | 0.1239 | -3.00302 |
| TCGA-A6-6141-01 | 0.3352 | -3.00372 |
| TCGA-AY-A71X-01 | 0.4814 | -3.00412 |
| TCGA-DY-A1DC-01 | 0.5887 | -3.00632 |
| TCGA-F4-6854-01 | 0.13275 | -3.01142 |
| TCGA-CM-6674-01 | 0.1541 | -3.01612 |
| TCGA-EF-5830-01 | 0.226 | -3.02372 |
| TCGA-G4-6306-01 | 0.3873 | -3.05742 |
| TCGA-AF-6672-01 | 0.1814 | -3.07682 |
| TCGA-G4-6321-01 | 0.476 | -3.08382 |
| TCGA-A6-6137-01 | 0.1892 | -3.10122 |
| TCGA-CM-6677-01 | 0.485 | -3.10962 |
| TCGA-G4-6303-01 | 0.2128 | -3.11992 |
| TCGA-AA-3511-01 | 0.32795 | -3.12222 |
| TCGA-CK-4948-01 | 0.0775 | -3.14822 |
| TCGA-CL-4957-01 | 0.30945 | -3.15032 |
| TCGA-AM-5820-01 | 0.49545 | -3.15192 |
| TCGA-G4-6323-01 | 0.38775 | -3.16432 |
| TCGA-5M-AATA-01 | 0.3937 | -3.17742 |
| TCGA-G5-6233-01 | 0.2086 | -3.19662 |
| TCGA-DM-A282-01 | 0.06435 | -3.20632 |
| TCGA-EI-6510-01 | 0.485 | -3.20692 |
| TCGA-A6-A56B-01 | 0.5994 | -3.22182 |
| TCGA-DM-A1D9-01 | 0.38565 | -3.23882 |
| TCGA-AM-5821-01 | 0.45285 | -3.26682 |
| TCGA-AA-3663-01 | 0.54385 | -3.26782 |
| TCGA-4N-A93T-01 | 0.63245 | -3.27402 |
| TCGA-D5-6538-01 | 0.89515 | -3.27492 |
| TCGA-D5-6924-01 | 0.38065 | -3.27512 |
| TCGA-AA-3655-01 | 0.07735 | -3.28062 |
| TCGA-CM-6172-01 | 0.41355 | -3.28472 |
| TCGA-5M-AATE-01 | 0.32535 | -3.29782 |
| TCGA-NH-A8F7-01 | 0.3391 | -3.32272 |
| TCGA-F4-6806-01 | 0.2789 | -3.33062 |
| TCGA-DM-A1DB-01 | 0.47175 | -3.33212 |
| TCGA-DC-6681-01 | 0.4199 | -3.33432 |
| TCGA-AA-3495-01 | 0.3322 | -3.33472 |
| TCGA-G4-6307-01 | 0.20745 | -3.33592 |
| TCGA-D5-6529-01 | 0.41945 | -3.34072 |
| TCGA-DM-A0X9-01 | 0.61905 | -3.34962 |
| TCGA-A6-5667-01 | 0.5263 | -3.35132 |
| TCGA-CI-6622-01 | 0.1453 | -3.35142 |
| TCGA-F4-6570-01 | 0.54295 | -3.35252 |
| TCGA-NH-A6GC-01 | 0.11585 | -3.36222 |
| TCGA-DM-A28E-01 | 0.30625 | -3.37342 |
| TCGA-CA-6716-01 | 0.371 | -3.39852 |
| TCGA-AY-A54L-01 | 0.5496 | -3.39922 |
| TCGA-5M-AAT5-01 | 0.282 | -3.40782 |
| TCGA-AF-2693-01 | 0.17035 | -3.42242 |
| TCGA-DM-A0XD-01 | 0.4554 | -3.42782 |
| TCGA-QG-A5YW-01 | 0.42755 | -3.43052 |
| TCGA-D5-6539-01 | 0.25975 | -3.43272 |
| TCGA-CM-6161-01 | 0.50505 | -3.43622 |
| TCGA-AA-3660-01 | 0.5681 | -3.43902 |
| TCGA-D5-5540-01 | 0.46935 | -3.44232 |
| TCGA-G4-6311-01 | 0.09735 | -3.45022 |
| TCGA-DM-A28A-01 | 0.5619 | -3.45532 |
| TCGA-EI-6511-01 | 0.40215 | -3.45802 |
| TCGA-A6-6648-01 | 0.25565 | -3.46112 |
| TCGA-D5-6530-01 | 0.3253 | -3.46422 |
| TCGA-AG-3591-01 | 0.1947 | -3.47652 |
| TCGA-CM-5863-01 | 0.33765 | -3.50382 |
| TCGA-EI-6508-01 | 0.3896 | -3.50772 |
| TCGA-DC-6160-01 | 0.16615 | -3.50962 |
| TCGA-QG-A5Z1-01 | 0.55015 | -3.52232 |
| TCGA-AA-3509-01 | 0.44845 | -3.52592 |
| TCGA-CM-5864-01 | 0.30775 | -3.54962 |
| TCGA-AA-3713-01 | 0.4299 | -3.57292 |
| TCGA-DC-6157-01 | 0.09475 | -3.57852 |
| TCGA-D5-6932-01 | 0.25895 | -3.58782 |
| TCGA-WS-AB45-01 | 0.3656 | -3.62392 |
| TCGA-AH-6544-01 | 0.5468 | -3.63732 |
| TCGA-DM-A285-01 | 0.236 | -3.64732 |
| TCGA-D5-6931-01 | 0.47725 | -3.66512 |
| TCGA-CK-4951-01 | 0.47485 | -3.68032 |
| TCGA-G4-6310-01 | 0.3197 | -3.71802 |
| TCGA-AF-A56N-01 | 0.14535 | -3.73042 |
| TCGA-AG-3725-01 | 0.065 | -3.73452 |
| TCGA-AD-A5EJ-01 | 0.6162 | -3.74872 |
| TCGA-D5-6535-01 | 0.4827 | -3.74912 |
| TCGA-AA-3502-01 | 0.3936 | -3.76002 |
| TCGA-D5-6533-01 | 0.17915 | -3.76472 |
| TCGA-DM-A1D8-01 | 0.4065 | -3.77592 |
| TCGA-D5-6537-01 | 0.24835 | -3.78252 |
| TCGA-DC-6155-01 | 0.42655 | -3.82692 |
| TCGA-QG-A5Z2-01 | 0.42625 | -3.83132 |
| TCGA-A6-5661-01 | 0.503625 | -3.83722 |
| TCGA-CK-4947-01 | 0.3989 | -3.86252 |
| TCGA-EI-6881-01 | 0.1466 | -3.88902 |
| TCGA-AG-3742-01 | 0.11935 | -3.89772 |
| TCGA-G4-6295-01 | 0.07965 | -3.90382 |
| TCGA-AY-6197-01 | 0.6017 | -3.90512 |
| TCGA-AD-A5EK-01 | 0.5644 | -3.92252 |
| TCGA-F5-6861-01 | 0.3975 | -3.92992 |
| TCGA-EF-5831-01 | 0.38925 | -3.93512 |
| TCGA-A6-6652-01 | 0.10335 | -3.95672 |
| TCGA-EI-6882-01 | 0.3571 | -3.96132 |
| TCGA-AA-3697-01 | 0.505 | -3.96172 |
| TCGA-AY-A8YK-01 | 0.16715 | -3.96172 |
| TCGA-EI-7002-01 | 0.4504 | -3.96232 |
| TCGA-DM-A28C-01 | 0.04195 | -3.96352 |
| TCGA-AZ-4682-01 | 0.06255 | -3.97182 |
| TCGA-CM-6166-01 | 0.19385 | -4.00422 |
| TCGA-AZ-6599-01 | 0.71195 | -4.01352 |
| TCGA-A6-5666-01 | 0.50645 | -4.01372 |
| TCGA-DM-A0XF-01 | 0.39395 | -4.01612 |
| TCGA-D5-7000-01 | 0.27615 | -4.02712 |
| TCGA-EI-6506-01 | 0.319 | -4.03652 |
| TCGA-AF-6136-01 | 0.10095 | -4.05372 |
| TCGA-CK-5914-01 | 0.48495 | -4.05812 |
| TCGA-G4-6322-01 | 0.65155 | -4.06222 |
| TCGA-CI-6623-01 | 0.24105 | -4.09082 |
| TCGA-A6-5656-01 | 0.42455 | -4.10432 |
| TCGA-DC-4749-01 | 0.0524 | -4.10542 |
| TCGA-F4-6808-01 | 0.13405 | -4.12802 |
| TCGA-CK-5912-01 | 0.04575 | -4.13292 |
| TCGA-CK-4950-01 | 0.376 | -4.15682 |
| TCGA-AA-3492-01 | 0.69375 | -4.15912 |
| TCGA-D5-6920-01 | 0.2873 | -4.16962 |
| TCGA-AU-3779-01 | 0.17135 | -4.17832 |
| TCGA-G4-6309-01 | 0.22455 | -4.18352 |
| TCGA-A6-6650-01 | 0.08580015 | -4.18722 |
| TCGA-G5-6641-01 | 0.3702 | -4.19072 |
| TCGA-AZ-4315-01 | 0.2498 | -4.19192 |
| TCGA-AD-6895-01 | 0.56375 | -4.20142 |
| TCGA-AZ-4615-01 | 0.36475 | -4.20462 |
| TCGA-NH-A50U-01 | 0.19245 | -4.20722 |
| TCGA-EI-6512-01 | 0.57885 | -4.21382 |
| TCGA-D5-5537-01 | 0.24515 | -4.24272 |
| TCGA-AY-5543-01 | 0.09795 | -4.24762 |
| TCGA-AD-6890-01 | 0.0844 | -4.24862 |
| TCGA-DY-A1DG-01 | 0.3966 | -4.25382 |
| TCGA-4T-AA8H-01 | 0.3557 | -4.26142 |
| TCGA-AZ-6606-01 | 0.68765 | -4.26712 |
| TCGA-A6-6140-01 | 0.22485 | -4.30192 |
| TCGA-AD-6889-01 | 0.7873 | -4.30782 |
| TCGA-G5-6235-01 | 0.151 | -4.34522 |
| TCGA-RU-A8FL-01 | 0.6079 | -4.34812 |
| TCGA-AU-6004-01 | 0.40555 | -4.39402 |
| TCGA-DM-A28F-01 | 0.30945 | -4.39882 |
| TCGA-AD-6888-01 | 0.1927 | -4.40532 |
| TCGA-QG-A5YV-01 | 0.5269 | -4.42462 |
| TCGA-AA-3496-01 | 0.29975 | -4.46012 |
| TCGA-CL-5918-01 | 0.20535 | -4.46682 |
| TCGA-D5-6540-01 | 0.57755 | -4.52862 |
| TCGA-T9-A92H-01 | 0.368 | -4.54482 |
| TCGA-CK-6751-01 | 0.43495 | -4.56332 |
| TCGA-DM-A1D4-01 | 0.11935 | -4.58202 |
| TCGA-DM-A288-01 | 0.2551 | -4.58322 |
| TCGA-CA-5255-01 | 0.39935 | -4.59382 |
| TCGA-CK-6747-01 | 0.1974 | -4.60222 |
| TCGA-QL-A97D-01 | 0.1997 | -4.64462 |
| TCGA-G4-6320-01 | 0.6124 | -4.64942 |
| TCGA-CA-5256-01 | 0.40735 | -4.65142 |
| TCGA-CK-5913-01 | 0.61195 | -4.65682 |
| TCGA-F4-6856-01 | 0.288 | -4.66062 |
| TCGA-AZ-4614-01 | 0.5896 | -4.69112 |
| TCGA-AZ-5407-01 | 0.5917 | -4.69622 |
| TCGA-CM-6675-01 | 0.8044 | -4.70692 |
| TCGA-A6-6780-01 | 0.3067165 | -4.72862 |
| TCGA-NH-A6GB-01 | 0.4541 | -4.80282 |
| TCGA-DM-A28K-01 | 0.13175 | -4.82612 |
| TCGA-CM-6171-01 | 0.5372 | -4.83592 |
| TCGA-AY-6386-01 | 0.5177 | -4.84752 |
| TCGA-CM-5861-01 | 0.4958 | -4.85662 |
| TCGA-G4-6304-01 | 0.3351 | -4.86952 |
| TCGA-G4-6317-01 | 0.67725 | -4.94072 |
| TCGA-CK-4952-01 | 0.40315 | -4.94152 |
| TCGA-CM-4743-01 | 0.33975 | -4.99702 |
| TCGA-CA-6715-01 | 0.14 | -5.05312 |
| TCGA-AZ-4313-01 | 0.0495 | -5.09742 |
| TCGA-DM-A1HB-01 | 0.51795 | -5.10342 |
| TCGA-DC-5337-01 | 0.3565 | -5.10952 |
| TCGA-CM-4744-01 | 0.45315 | -5.14772 |
| TCGA-G4-6293-01 | 0.23035 | -5.16902 |
| TCGA-D5-6532-01 | 0.70155 | -5.17492 |
| TCGA-G4-6586-01 | 0.4813 | -5.20862 |
| TCGA-AA-3712-01 | 0.2006 | -5.21772 |
| TCGA-G4-6588-01 | 0.4717 | -5.22842 |
| TCGA-AD-6963-01 | 0.117 | -5.24782 |
| TCGA-AH-6897-01 | 0.0713 | -5.29592 |
| TCGA-CK-6746-01 | 0.55475 | -5.32322 |
| TCGA-NH-A50T-01 | 0.1436 | -5.33972 |
| TCGA-AZ-6598-01 | 0.6069 | -5.37752 |
| TCGA-G4-6315-01 | 0.38045 | -5.42542 |
| TCGA-AZ-6608-01 | 0.08135 | -5.43282 |
| TCGA-AH-6903-01 | 0.30785 | -5.49712 |
| TCGA-A6-5665-01 | 0.590825 | -5.51302 |
| TCGA-DM-A28M-01 | 0.16485 | -5.54252 |
| TCGA-DM-A280-01 | 0.1874 | -5.60332 |
| TCGA-CK-5915-01 | 0.3756 | -5.81572 |
| TCGA-QG-A5YX-01 | 0.31035 | -5.88192 |
| TCGA-A6-6653-01 | 0.74825 | -6.03072 |
| TCGA-DM-A1DA-01 | 0.64315 | -6.32692 |
| TCGA-CA-5796-01 | 0.33195 | -7.03732 |
