# Supplementary material for: Methylated claudin-11 associated with metastasis and poor survival of colorectal cancer
Source: Oncotarget. 2017 Oct 23;8(56):96249–62. doi: 10.18632/oncotarget.21997 (PMC5707097; doi:10.18632/oncotarget.21997)
Supplement: Supplementary file 4 [file oncotarget-08-96249-s004.docx]

**Supplementary Table 3: Progression free survival (RFS) data of 339 CRC patients from TCGA**

| Sample | RFS time (days) | RFS status | Group |
| --- | --- | --- | --- |
| TCGA-G4-6323-01 | 419 | 1 | Hypermethylation |
| TCGA-A6-2682-11 | 381 | 1 | Hypomethylation |
| TCGA-A6-2682-01 | 381 | 1 | Hypermethylation |
| TCGA-D5-6931-01 | 365 | 1 | Hypermethylation |
| TCGA-A6-6781-01 | 598 | 1 | Hypermethylation |
| TCGA-A6-5666-01 | 970 | 1 | Hypermethylation |
| TCGA-AF-4110-01 | 912 | 1 | Hypermethylation |
| TCGA-DC-6156-01 | 30 | 1 | Hypermethylation |
| TCGA-CM-4747-01 | 761 | 1 | Hypomethylation |
| TCGA-AF-6672-01 | 461 | 1 | Hypomethylation |
| TCGA-NH-A8F8-01 | 511 | 1 | Hypermethylation |
| TCGA-AD-6899-01 | 176 | 1 | Hypomethylation |
| TCGA-AD-6965-01 | 648 | 1 | Hypomethylation |
| TCGA-A6-5664-01 | 643 | 1 | Hypermethylation |
| TCGA-NH-A6GA-01 | 6 | 1 | Hypomethylation |
| TCGA-F5-6813-01 | 65 | 1 | Hypermethylation |
| TCGA-4N-A93T-01 | 146 | 1 | Hypermethylation |
| TCGA-CM-4748-01 | 792 | 1 | Hypomethylation |
| TCGA-G4-6298-11 | 291 | 1 | Hypomethylation |
| TCGA-G4-6298-01 | 291 | 1 | Hypomethylation |
| TCGA-D5-6926-01 | 275 | 1 | Hypomethylation |
| TCGA-A6-6782-01 | 617 | 1 | Hypermethylation |
| TCGA-EF-5830-01 | 106 | 1 | Hypomethylation |
| TCGA-AD-6964-01 | 325 | 1 | Hypomethylation |
| TCGA-AZ-6606-01 | 357 | 1 | Hypermethylation |
| TCGA-AZ-6607-01 | 97 | 1 | Hypermethylation |
| TCGA-AZ-4323-01 | 43 | 1 | Hypermethylation |
| TCGA-AZ-6605-01 | 159 | 1 | Hypermethylation |
| TCGA-AA-3511-01 | 59 | 1 | Hypermethylation |
| TCGA-G5-6233-01 | 92 | 1 | Hypomethylation |
| TCGA-CM-6675-01 | 337 | 1 | Hypermethylation |
| TCGA-AY-A8YK-01 | 573 | 1 | Hypomethylation |
| TCGA-A6-5662-01 | 661 | 1 | Hypomethylation |
| TCGA-AZ-4684-01 | 411 | 1 | Hypomethylation |
| TCGA-CM-5862-01 | 153 | 1 | Hypermethylation |
| TCGA-F5-6702-01 | 368 | 1 | Hypomethylation |
| TCGA-DY-A1DG-01 | 592 | 1 | Hypermethylation |
| TCGA-A6-6648-01 | 686 | 1 | Hypomethylation |
| TCGA-A6-6652-01 | 751 | 1 | Hypomethylation |
| TCGA-AZ-4682-01 | 680 | 1 | Hypomethylation |
| TCGA-A6-2671-01 | 1215 | 1 | Hypermethylation |
| TCGA-A6-2671-11 | 1215 | 1 | Hypomethylation |
| TCGA-EI-7002-01 | 364 | 1 | Hypermethylation |
| TCGA-A6-A567-01 | 1069 | 1 | Hypomethylation |
| TCGA-G4-6315-01 | 1883 | 1 | Hypermethylation |
| TCGA-G4-6294-01 | 517 | 1 | Hypermethylation |
| TCGA-AG-3731-11 | 1126 | 1 | Hypomethylation |
| TCGA-AG-3731-01 | 112 | 1 | Hypermethylation |
| TCGA-5M-AAT4-01 | 49 | 1 | Hypomethylation |
| TCGA-AA-3713-11 | 579 | 1 | Hypomethylation |
| TCGA-AA-3713-01 | 579 | 1 | Hypermethylation |
| TCGA-A6-5660-01 | 888 | 1 | Hypomethylation |
| TCGA-EI-6509-01 | 90 | 1 | Hypomethylation |
| TCGA-AH-6643-01 | 1026 | 1 | Hypermethylation |
| TCGA-AF-3911-01 | 1020 | 1 | Hypomethylation |
| TCGA-AF-2687-01 | 1267 | 1 | Hypermethylation |
| TCGA-G4-6299-01 | 2268 | 1 | Hypermethylation |
| TCGA-DM-A28H-01 | 484 | 1 | Hypermethylation |
| TCGA-DM-A28A-01 | 805 | 1 | Hypermethylation |
| TCGA-DM-A288-01 | 320 | 1 | Hypomethylation |
| TCGA-DM-A1HA-01 | 400 | 1 | Hypermethylation |
| TCGA-RU-A8FL-01 | 256 | 1 | Hypermethylation |
| TCGA-NH-A50V-01 | 588 | 1 | Hypermethylation |
| TCGA-A6-6137-01 | 824 | 1 | Hypomethylation |
| TCGA-QG-A5Z1-01 | 256 | 1 | Hypermethylation |
| TCGA-CM-4751-01 | 822 | 1 | Hypermethylation |
| TCGA-A6-6649-01 | 735 | 1 | Hypermethylation |
| TCGA-AD-6888-01 | 343 | 1 | Hypomethylation |
| TCGA-DC-6683-01 | 762 | 1 | Hypermethylation |
| TCGA-AH-6903-01 | 592 | 1 | Hypomethylation |
| TCGA-D5-6537-01 | 123 | 1 | Hypomethylation |
| TCGA-AD-6895-01 | 763 | 1 | Hypermethylation |
| TCGA-CM-5348-01 | 699 | 1 | Hypomethylation |
| TCGA-D5-5541-01 | 1701 | 1 | Hypermethylation |
| TCGA-G4-6322-11 | 789 | 1 | Hypomethylation |
| TCGA-G4-6322-01 | 789 | 1 | Hypermethylation |
| TCGA-G5-6235-01 | 1042 | 1 | Hypomethylation |
| TCGA-A6-A56B-01 | 1678 | 0 | Hypermethylation |
| TCGA-A6-5657-01 | 962 | 0 | Hypermethylation |
| TCGA-A6-A5ZU-01 | 293 | 0 | Hypermethylation |
| TCGA-AZ-4615-01 | 1002 | 0 | Hypermethylation |
| TCGA-EI-6511-01 | 482 | 0 | Hypermethylation |
| TCGA-G4-6310-01 | 1935 | 0 | Hypermethylation |
| TCGA-DM-A1HB-01 | 4126 | 0 | Hypermethylation |
| TCGA-EI-6513-01 | 497 | 0 | Hypomethylation |
| TCGA-CA-6715-01 | 383 | 0 | Hypomethylation |
| TCGA-DM-A28F-01 | 1094 | 0 | Hypomethylation |
| TCGA-BM-6198-01 | 646 | 0 | Hypomethylation |
| TCGA-D5-5539-01 | 579 | 0 | Hypermethylation |
| TCGA-D5-6922-01 | 308 | 0 | Hypomethylation |
| TCGA-EI-6917-01 | 531 | 0 | Hypermethylation |
| TCGA-EI-6881-01 | 499 | 0 | Hypomethylation |
| TCGA-EI-6884-01 | 328 | 0 | Hypermethylation |
| TCGA-AG-A036-11 | 3562 | 0 | Hypomethylation |
| TCGA-AG-A036-01 | 3562 | 0 | Hypermethylation |
| TCGA-G4-6311-11 | 1199 | 0 | Hypomethylation |
| TCGA-G4-6311-01 | 1199 | 0 | Hypomethylation |
| TCGA-G4-6320-11 | 804 | 0 | Hypomethylation |
| TCGA-G4-6320-01 | 804 | 0 | Hypermethylation |
| TCGA-EI-6883-01 | 350 | 0 | Hypermethylation |
| TCGA-D5-5537-01 | 722 | 0 | Hypomethylation |
| TCGA-A6-2675-01 | 1321 | 0 | Hypomethylation |
| TCGA-A6-2675-11 | 1321 | 0 | Hypomethylation |
| TCGA-A6-6780-01 | 612 | 0 | Hypomethylation |
| TCGA-CM-4743-01 | 701 | 0 | Hypermethylation |
| TCGA-CM-6169-01 | 396 | 0 | Hypomethylation |
| TCGA-AF-A56K-01 | 2635 | 0 | Hypomethylation |
| TCGA-G4-6627-01 | 2275 | 0 | Hypomethylation |
| TCGA-AD-6889-01 | 2532 | 0 | Hypermethylation |
| TCGA-D5-5540-01 | 1706 | 0 | Hypermethylation |
| TCGA-F4-6703-01 | 1456 | 0 | Hypomethylation |
| TCGA-5M-AATE-01 | 810 | 0 | Hypermethylation |
| TCGA-F5-6814-01 | 1131 | 0 | Hypermethylation |
| TCGA-A6-3810-01 | 1111 | 0 | Hypomethylation |
| TCGA-F5-6812-01 | 1110 | 0 | Hypomethylation |
| TCGA-F4-6463-01 | 1087 | 0 | Hypermethylation |
| TCGA-AG-3592-01 | 1035 | 0 | Hypomethylation |
| TCGA-CM-5860-01 | 974 | 0 | Hypermethylation |
| TCGA-DC-6682-01 | 762 | 0 | Hypermethylation |
| TCGA-A6-6140-01 | 734 | 0 | Hypomethylation |
| TCGA-D5-6930-01 | 406 | 0 | Hypermethylation |
| TCGA-CM-4752-01 | 396 | 0 | Hypomethylation |
| TCGA-T9-A92H-01 | 81 | 0 | Hypermethylation |
| TCGA-DM-A28K-01 | 2988 | 0 | Hypomethylation |
| TCGA-DM-A28M-01 | 2895 | 0 | Hypomethylation |
| TCGA-DM-A1D4-01 | 1920 | 0 | Hypomethylation |
| TCGA-DM-A28G-01 | 1849 | 0 | Hypomethylation |
| TCGA-AZ-4315-01 | 1776 | 0 | Hypomethylation |
| TCGA-DM-A1D6-01 | 518 | 0 | Hypermethylation |
| TCGA-DM-A1DB-01 | 348 | 0 | Hypermethylation |
| TCGA-D5-6529-01 | 386 | 0 | Hypermethylation |
| TCGA-EI-6507-01 | 607 | 0 | Hypermethylation |
| TCGA-D5-6532-01 | 555 | 0 | Hypermethylation |
| TCGA-D5-6536-01 | 493 | 0 | Hypomethylation |
| TCGA-D5-6531-01 | 540 | 0 | Hypermethylation |
| TCGA-CM-6165-01 | 488 | 0 | Hypermethylation |
| TCGA-D5-6541-01 | 474 | 0 | Hypomethylation |
| TCGA-D5-6924-01 | 435 | 0 | Hypermethylation |
| TCGA-DM-A1D7-01 | 154 | 0 | Hypomethylation |
| TCGA-CM-6674-01 | 394 | 0 | Hypomethylation |
| TCGA-D5-6928-01 | 354 | 0 | Hypomethylation |
| TCGA-D5-6932-01 | 346 | 0 | Hypomethylation |
| TCGA-CM-6679-01 | 306 | 0 | Hypermethylation |
| TCGA-EI-6882-01 | 262 | 0 | Hypermethylation |
| TCGA-G4-6626-01 | 1422 | 0 | Hypomethylation |
| TCGA-DM-A0XD-01 | 617 | 0 | Hypermethylation |
| TCGA-A6-4105-01 | 364 | 0 | Hypermethylation |
| TCGA-EF-5831-01 | 127 | 0 | Hypermethylation |
| TCGA-CA-6719-01 | 354 | 0 | Hypermethylation |
| TCGA-CA-6717-01 | 388 | 0 | Hypermethylation |
| TCGA-CA-5797-01 | 383 | 0 | Hypomethylation |
| TCGA-CA-5255-01 | 376 | 0 | Hypermethylation |
| TCGA-CA-6716-01 | 371 | 0 | Hypermethylation |
| TCGA-CA-6718-01 | 207 | 0 | Hypermethylation |
| TCGA-DM-A28C-01 | 1929 | 0 | Hypomethylation |
| TCGA-AY-6197-01 | 652 | 0 | Hypermethylation |
| TCGA-AZ-5403-01 | 304 | 0 | Hypomethylation |
| TCGA-AZ-6601-11 | 2270 | 0 | Hypomethylation |
| TCGA-AZ-6601-01 | 2270 | 0 | Hypermethylation |
| TCGA-AA-3510-01 | 1946 | 0 | Hypermethylation |
| TCGA-AA-3510-11 | 1946 | 0 | Hypomethylation |
| TCGA-AA-3655-11 | 1856 | 0 | Hypomethylation |
| TCGA-AA-3655-01 | 1856 | 0 | Hypomethylation |
| TCGA-AA-3663-01 | 212 | 0 | Hypermethylation |
| TCGA-AA-3663-11 | 212 | 0 | Hypomethylation |
| TCGA-DT-5265-01 | 384 | 0 | Hypermethylation |
| TCGA-AH-6644-01 | 838 | 0 | Hypermethylation |
| TCGA-AH-6549-01 | 532 | 0 | Hypermethylation |
| TCGA-AH-6544-01 | 1173 | 0 | Hypermethylation |
| TCGA-AD-6963-01 | 834 | 0 | Hypomethylation |
| TCGA-AD-6901-01 | 468 | 0 | Hypermethylation |
| TCGA-G4-6306-01 | 1359 | 0 | Hypermethylation |
| TCGA-AF-6655-01 | 609 | 0 | Hypomethylation |
| TCGA-CK-5912-01 | 1493 | 0 | Hypomethylation |
| TCGA-AF-2693-01 | 1155 | 0 | Hypomethylation |
| TCGA-AD-5900-01 | 370 | 0 | Hypermethylation |
| TCGA-AZ-6599-11 | 206 | 0 | Hypomethylation |
| TCGA-AZ-6599-01 | 206 | 0 | Hypermethylation |
| TCGA-AD-A5EK-01 | 500 | 0 | Hypermethylation |
| TCGA-DC-4749-01 | 762 | 0 | Hypomethylation |
| TCGA-CM-4744-01 | 609 | 0 | Hypermethylation |
| TCGA-CM-6676-01 | 337 | 0 | Hypermethylation |
| TCGA-G4-6628-01 | 242 | 0 | Hypermethylation |
| TCGA-DC-6157-01 | 1581 | 0 | Hypomethylation |
| TCGA-DC-6160-01 | 1339 | 0 | Hypomethylation |
| TCGA-CM-4746-01 | 1126 | 0 | Hypermethylation |
| TCGA-F4-6569-01 | 87 | 0 | Hypermethylation |
| TCGA-F4-6856-01 | 1074 | 0 | Hypomethylation |
| TCGA-A6-5656-01 | 100 | 0 | Hypermethylation |
| TCGA-QG-A5Z2-01 | 952 | 0 | Hypermethylation |
| TCGA-A6-5659-01 | 926 | 0 | Hypermethylation |
| TCGA-AH-6897-01 | 804 | 0 | Hypomethylation |
| TCGA-A6-6138-01 | 685 | 0 | Hypomethylation |
| TCGA-D5-6530-01 | 621 | 0 | Hypermethylation |
| TCGA-D5-6540-01 | 491 | 0 | Hypermethylation |
| TCGA-CM-5864-01 | 457 | 0 | Hypomethylation |
| TCGA-D5-6923-01 | 378 | 0 | Hypomethylation |
| TCGA-DC-6158-01 | 334 | 0 | Hypomethylation |
| TCGA-AA-3495-11 | 1127 | 0 | Hypomethylation |
| TCGA-AA-3495-01 | 127 | 0 | Hypermethylation |
| TCGA-G5-6641-01 | 804 | 0 | Hypermethylation |
| TCGA-DC-5337-01 | 792 | 0 | Hypermethylation |
| TCGA-CM-6163-01 | 427 | 0 | Hypomethylation |
| TCGA-AD-6890-01 | 746 | 0 | Hypomethylation |
| TCGA-G5-6572-01 | 1432 | 0 | Hypomethylation |
| TCGA-G5-6572-02 | 1432 | 0 | Hypomethylation |
| TCGA-D5-6533-01 | 775 | 0 | Hypomethylation |
| TCGA-NH-A6GC-01 | 389 | 0 | Hypomethylation |
| TCGA-F5-6464-01 | 303 | 0 | Hypomethylation |
| TCGA-F4-6461-01 | 288 | 0 | Hypermethylation |
| TCGA-QG-A5YV-01 | 301 | 0 | Hypermethylation |
| TCGA-EI-7004-01 | 257 | 0 | Hypomethylation |
| TCGA-DC-6154-01 | 365 | 0 | Hypomethylation |
| TCGA-CM-5868-01 | 518 | 0 | Hypomethylation |
| TCGA-AZ-4614-01 | 172 | 0 | Hypermethylation |
| TCGA-5M-AAT6-01 | 219 | 0 | Hypomethylation |
| TCGA-F5-6863-01 | 191 | 0 | Hypomethylation |
| TCGA-SS-A7HO-01 | 801 | 0 | Hypermethylation |
| TCGA-AA-3662-01 | 184 | 0 | Hypomethylation |
| TCGA-A6-A566-01 | 257 | 0 | Hypermethylation |
| TCGA-A6-3809-01 | 996 | 0 | Hypermethylation |
| TCGA-G4-6304-01 | 859 | 0 | Hypermethylation |
| TCGA-D5-6539-01 | 380 | 0 | Hypomethylation |
| TCGA-DC-6681-01 | 790 | 0 | Hypermethylation |
| TCGA-A6-6142-01 | 654 | 0 | Hypermethylation |
| TCGA-AY-5543-01 | 1004 | 0 | Hypomethylation |
| TCGA-F4-6809-01 | 403 | 0 | Hypermethylation |
| TCGA-CI-6620-01 | 90 | 0 | Hypomethylation |
| TCGA-G4-6297-11 | 2394 | 0 | Hypomethylation |
| TCGA-G4-6297-01 | 394 | 0 | Hypermethylation |
| TCGA-G4-6314-01 | 38 | 0 | Hypomethylation |
| TCGA-G4-6314-11 | 38 | 0 | Hypomethylation |
| TCGA-AZ-4616-01 | 156 | 0 | Hypermethylation |
| TCGA-DM-A285-01 | 179 | 0 | Hypomethylation |
| TCGA-EI-6885-01 | 295 | 0 | Hypermethylation |
| TCGA-G4-6303-01 | 872 | 0 | Hypomethylation |
| TCGA-D5-6929-01 | 408 | 0 | Hypomethylation |
| TCGA-QG-A5YW-01 | 896 | 0 | Hypermethylation |
| TCGA-NH-A6GB-01 | 476 | 0 | Hypermethylation |
| TCGA-CM-6167-01 | 456 | 0 | Hypomethylation |
| TCGA-F4-6807-01 | 309 | 0 | Hypermethylation |
| TCGA-CL-5917-01 | 376 | 0 | Hypermethylation |
| TCGA-A6-A565-01 | 439 | 0 | Hypomethylation |
| TCGA-G4-6317-01 | 592 | 0 | Hypermethylation |
| TCGA-G4-6317-02 | 592 | 0 | Hypomethylation |
| TCGA-AF-A56L-01 | 200 | 0 | Hypermethylation |
| TCGA-A6-2677-01 | 740 | 0 | Hypomethylation |
| TCGA-DM-A0XF-01 | 162 | 0 | Hypermethylation |
| TCGA-DM-A1DA-01 | 228 | 0 | Hypermethylation |
| TCGA-CM-6680-01 | 366 | 0 | Hypermethylation |
| TCGA-F4-6459-01 | 262 | 0 | Hypermethylation |
| TCGA-D5-6538-01 | 521 | 0 | Hypermethylation |
| TCGA-AF-6136-01 | 609 | 0 | Hypomethylation |
| TCGA-A6-6651-01 | 662 | 0 | Hypermethylation |
| TCGA-A6-4107-01 | 987 | 0 | Hypermethylation |
| TCGA-A6-4107-11 | 987 | 0 | Hypomethylation |
| TCGA-CM-5344-01 | 670 | 0 | Hypermethylation |
| TCGA-D5-5538-01 | 1007 | 0 | Hypermethylation |
| TCGA-CM-5863-01 | 457 | 0 | Hypermethylation |
| TCGA-A6-5667-01 | 887 | 0 | Hypermethylation |
| TCGA-A6-5667-11 | 887 | 0 | Hypomethylation |
| TCGA-EI-6508-01 | 636 | 0 | Hypermethylation |
| TCGA-CM-6162-01 | 365 | 0 | Hypomethylation |
| TCGA-DC-5869-01 | 943 | 0 | Hypermethylation |
| TCGA-DC-4745-01 | 639 | 0 | Hypomethylation |
| TCGA-AY-6386-01 | 542 | 0 | Hypermethylation |
| TCGA-CM-6172-01 | 335 | 0 | Hypermethylation |
| TCGA-D5-6535-01 | 460 | 0 | Hypermethylation |
| TCGA-AZ-4308-01 | 3324 | 0 | Hypomethylation |
| TCGA-A6-2672-01 | 1419 | 0 | Hypermethylation |
| TCGA-A6-6654-01 | 726 | 0 | Hypermethylation |
| TCGA-G4-6307-01 | 1674 | 0 | Hypomethylation |
| TCGA-G4-6309-01 | 2176 | 0 | Hypomethylation |
| TCGA-CK-4950-01 | 2599 | 0 | Hypermethylation |
| TCGA-F4-6460-01 | 972 | 0 | Hypomethylation |
| TCGA-EI-6512-01 | 538 | 0 | Hypermethylation |
| TCGA-DY-A1DD-01 | 1741 | 0 | Hypomethylation |
| TCGA-AG-3725-11 | 638 | 0 | Hypomethylation |
| TCGA-AG-3725-01 | 638 | 0 | Hypomethylation |
| TCGA-G4-6293-01 | 4051 | 0 | Hypomethylation |
| TCGA-CK-4948-01 | 4502 | 0 | Hypomethylation |
| TCGA-A6-2679-11 | 1366 | 0 | Hypomethylation |
| TCGA-A6-2679-01 | 1366 | 0 | Hypermethylation |
| TCGA-CK-6747-01 | 2523 | 0 | Hypomethylation |
| TCGA-WS-AB45-01 | 213 | 0 | Hypermethylation |
| TCGA-CK-5913-01 | 156 | 0 | Hypermethylation |
| TCGA-QG-A5YX-01 | 1003 | 0 | Hypomethylation |
| TCGA-NH-A5IV-01 | 588 | 0 | Hypermethylation |
| TCGA-NH-A50T-01 | 553 | 0 | Hypomethylation |
| TCGA-NH-A8F7-01 | 543 | 0 | Hypermethylation |
| TCGA-NH-A8F7-06 | 543 | 0 | Hypomethylation |
| TCGA-4T-AA8H-01 | 385 | 0 | Hypermethylation |
| TCGA-CL-5918-01 | 218 | 0 | Hypomethylation |
| TCGA-A6-5665-01 | 529 | 0 | Hypermethylation |
| TCGA-G4-6302-11 | 2047 | 0 | Hypomethylation |
| TCGA-G4-6302-01 | 2047 | 0 | Hypomethylation |
| TCGA-G4-6586-01 | 1089 | 0 | Hypermethylation |
| TCGA-F5-6811-01 | 979 | 0 | Hypomethylation |
| TCGA-F4-6854-01 | 16 | 0 | Hypomethylation |
| TCGA-F5-6465-01 | 1506 | 0 | Hypomethylation |
| TCGA-F4-6855-01 | 44 | 0 | Hypermethylation |
| TCGA-A6-2681-01 | 1150 | 0 | Hypomethylation |
| TCGA-A6-2681-11 | 1150 | 0 | Hypomethylation |
| TCGA-D5-6534-01 | 1316 | 0 | Hypomethylation |
| TCGA-F5-6571-01 | 1059 | 0 | Hypermethylation |
| TCGA-F5-6861-01 | 116 | 0 | Hypermethylation |
| TCGA-A6-2685-11 | 948 | 0 | Hypomethylation |
| TCGA-A6-2685-01 | 948 | 0 | Hypomethylation |
| TCGA-F4-6805-01 | 1047 | 0 | Hypomethylation |
| TCGA-AG-3591-01 | 1035 | 0 | Hypomethylation |
| TCGA-A6-5661-01 | 1020 | 0 | Hypermethylation |
| TCGA-CM-5349-01 | 915 | 0 | Hypermethylation |
| TCGA-A6-6650-01 | 627 | 0 | Hypomethylation |
| TCGA-AY-A69D-01 | 543 | 0 | Hypomethylation |
| TCGA-A6-2686-01 | 112 | 0 | Hypermethylation |
| TCGA-A6-2686-11 | 1126 | 0 | Hypomethylation |
| TCGA-AF-A56N-01 | 360 | 0 | Hypomethylation |
| TCGA-DM-A1D9-01 | 427 | 0 | Hypermethylation |
| TCGA-DM-A282-01 | 4233 | 0 | Hypomethylation |
| TCGA-DM-A1D0-01 | 3974 | 0 | Hypomethylation |
| TCGA-DY-A1DE-01 | 3316 | 0 | Hypomethylation |
| TCGA-DY-A0XA-01 | 3846 | 0 | Hypomethylation |
| TCGA-DM-A28E-01 | 3648 | 0 | Hypomethylation |
| TCGA-DM-A0X9-01 | 364 | 0 | Hypermethylation |
| TCGA-G4-6625-01 | 2564 | 0 | Hypomethylation |
| TCGA-G4-6625-11 | 2564 | 0 | Hypomethylation |
| TCGA-CK-4951-01 | 480 | 0 | Hypermethylation |
| TCGA-CM-6164-01 | 883 | 0 | Hypomethylation |
| TCGA-G4-6588-01 | 796 | 0 | Hypermethylation |
| TCGA-EI-6506-01 | 625 | 0 | Hypermethylation |
| TCGA-EI-6514-01 | 496 | 0 | Hypomethylation |
| TCGA-CM-5861-01 | 457 | 0 | Hypermethylation |
| TCGA-CM-6168-01 | 395 | 0 | Hypomethylation |
| TCGA-D5-6920-01 | 377 | 0 | Hypomethylation |
| TCGA-CM-6677-01 | 337 | 0 | Hypermethylation |
| TCGA-DM-A280-01 | 236 | 0 | Hypomethylation |
| TCGA-F4-6570-01 | 188 | 0 | Hypermethylation |
| TCGA-DY-A1DC-01 | 630 | 0 | Hypermethylation |
| TCGA-AZ-4681-01 | 3247 | 0 | Hypomethylation |
